# Zeolite water purification at Tikal, an ancient Maya city in Guatemala

**DOI:** 10.1038/s41598-020-75023-7

**Published:** 2020-10-22

**Authors:** Kenneth Barnett Tankersley, Nicholas P. Dunning, Christopher Carr, David L. Lentz, Vernon L. Scarborough

**Affiliations:** 1 Department of Anthropology, University of Cincinnati, Cincinnati, OH 45221 USA; 2 Department of Geology, University of Cincinnati, Cincinnati, OH 45221 USA; 3 Department of Geography and GIS, University of Cincinnati, Cincinnati, OH 45221 USA; 4 Department of Biological Sciences, University of Cincinnati, Cincinnati, OH 45221 USA

**Keywords:** Environmental social sciences, Sustainability, Natural hazards

## Abstract

Evidence for the oldest known zeolite water purification filtration system occurs in the undisturbed sediments of the Corriental reservoir at the Maya city of Tikal, in northern Guatemala. The Corriental reservoir was an important source of drinking water at Tikal during the Late Preclassic to Late Classic cultural periods. X-ray diffraction analysis (XRD) and six AMS radiocarbon ages show that between ~ 2185 and 965 cal yr B.P. the drinking water in the Corriental reservoir water was filtered through a mixture of zeolite and coarse, sand-sized crystalline quartz. Zeolite is a non-toxic, three-dimensionally porous, crystalline, hydrated aluminosilicate with natural adsorbent and ion exchange properties, which removes harmful microbes as well as dispersed insoluble and soluble toxins from drinking water. The occurrence of zeolite in Corriental reservoir sediments expands our understanding of the earliest history of water purification and the long-term sustainability of an ancient Maya city.

## Introduction

Zeolite has long been recognized as a mineral with excellent absorptive properties^1^. Approximately 2700 years ago, Greek and Roman engineers used zeolites as a pozzolan in cement in the construction of large scale hydraulic structures such as aqueducts, bridges, dams, and harbors^2^. However, it has been assumed that zeolites were not used for water purification until the beginning of the twentieth century. It also has been presumed that the oldest forms of water purification occurred in Europe and southern Asia^3^.

Archaeologists have long believed that the Indigenous people of the Western Hemisphere lacked any formal water filtration systems. In North America, ancient Indigenous cultures obtained clean water from naturally filtered springs, used boiling, and earthenware pottery in which contaminants, silt, and clay were pulled to the sides of the vessel^3,4^. In Mesoamerica, the Aztec relied on abundant artesian spring water brought into their cities (e.g., Tenochtitlan) through aqueducts, which did not require purification techniques^5^. Aqueducts were also built by the Inca, which brought mountain spring water to cities in the Andean region of South America^6^. The Maya were the only ancient New World civilization that needed water filtration because many of their cities were located on a karst landscape in a tropical and monsoon climate. While sand, gravel, plant, and cloth filtration systems have been documented in Egypt, Greece, and South Asia as early as the fifteenth century BCE, comparable data are lacking for the Maya region. There is no comparative case for Maya water purification systems, that is, there are no comparative data available. To date, excavations have been conducted in only a few dozen of the many thousands of ancient Maya reservoirs, and many of these excavations have been limited to a single test pit. Here we report our findings from Tikal, Guatemala, where zeolite was found in the one of the largest storage facilities of Maya drinking water in use during the Late Preclassic to Late Classic cultural periods (~ 2200–1100 yr. B.P.). The apparent zeolite filtration system at Tikal’s Corriental reservoir is the oldest known example of water purification in the Western Hemisphere and the oldest known use of zeolite for decontaminating drinking water in the world.

### Site setting

**Figure 1:**
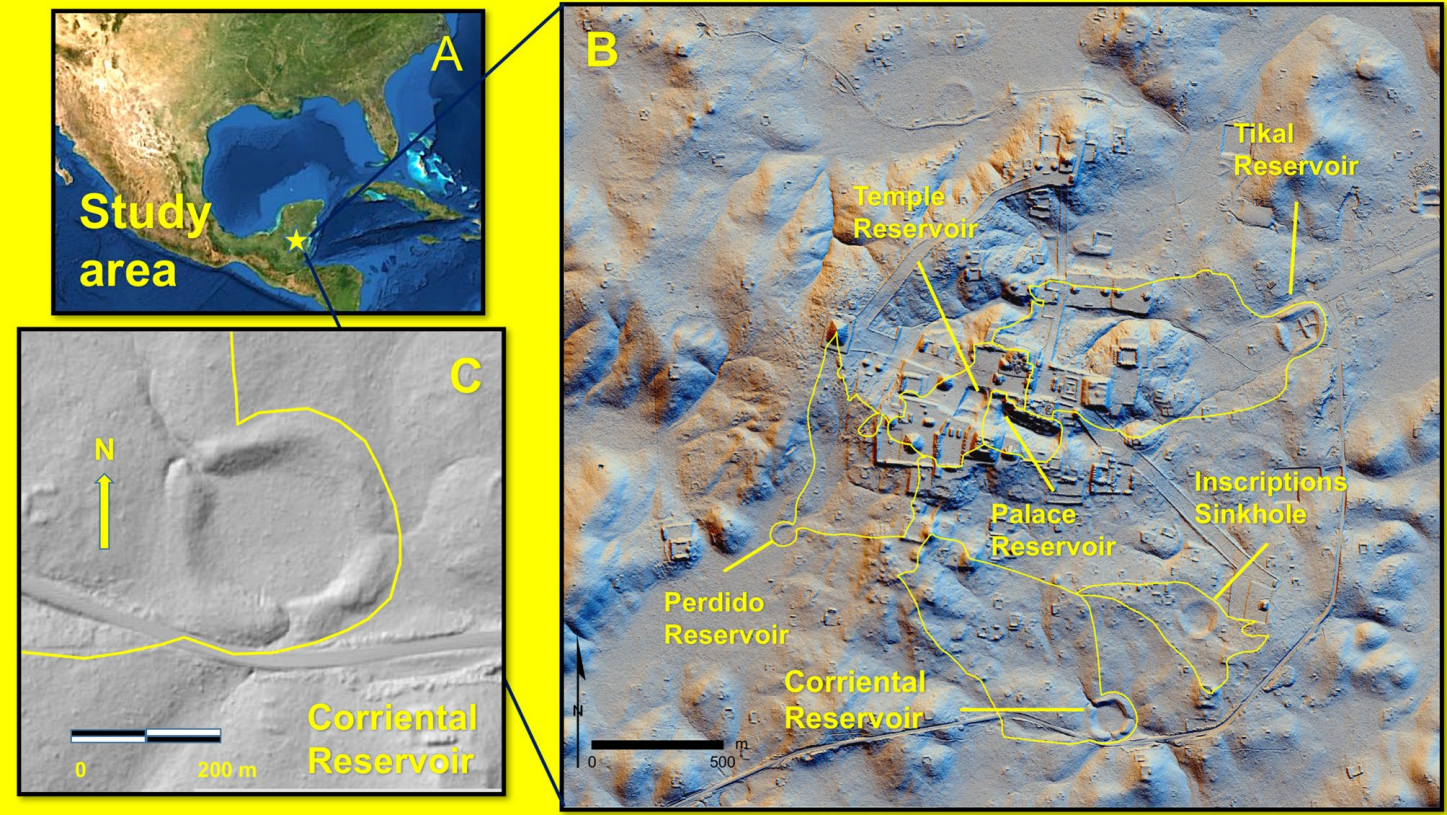
Tikal site map. (**A**) Location of Tikal in the southern Maya lowland. (**B**) The location of the Corriental, Palace, Perdido, Temple, and Tikal reservoirs, and the Inscription sinkhole and their catchment areas. (**C**) A lidar-derived hillshade image of the Corriental reservoir. The lidar-derived hillshade images (**B**,**C**) were created by Francisco Estrada-Belli, a principle of the PACUNAM Lidar initiative^23^. Christopher Carr used ESRI ArcGIS 10.3.1 software (www.esri.com) to create a GIS layer of the catchment areas, georeferenced on the hillshades to make the base maps. Kenneth Barnett Tankersley then used Microsoft PowerPoint for Mac Version 16.41 (www.microsoft.com) to create this figure.

Tikal, known as Yax Mutal to the ancient Maya, was a city of more than 3000 structures situated in the karst topography and tropical forest ecosystem of the southern Maya Lowlands (Fig. 1A,B). Throughout the late Holocene, this area was affected by highly variable seasonal precipitation and subsurface drainage^7^. In this environmental setting, the Maya constructed reservoirs to provide a reliable and sustainable source of drinking water during both seasonal and cyclical droughts^8^. The Maya rulers of Tikal closely associated themselves with the provision of clean water^9,10^.

The first systematic archaeological investigation of Tikal began in 1881 and accelerated through the twentieth century, resulting in detailed maps, photographs, and stratigraphic records of the site^7^ (Section S1). While subsequent investigations documented the architecture and planning of Tikal’s water management system, including precise dimensions of the catchment areas, human-modified watersheds, reservoirs, and their carrying capacity, the issue of water purification remained unresolved^8^. Given the area is subject to extreme seasonal droughts, a large population, and long-term occupation, the drinking water of Tikal was prone to contamination from a plethora of microbial sources (e.g., cyanobacteria) and leachates from toxic minerals such as cinnabar^11^. Uncertainty remains about how these contaminants might have been removed from the drinking water. We reexamined three of the largest reservoirs at Tikal and a sinkhole as a control to evaluate the mineralogical composition and chronological contexts of the sediments in order to assess potential water purification methods and their time of implementation.

## Results

### Mineralogical and chronological context

Our mineralogical and chronological analyses focused on three reservoirs (Corriental, Perdido, and Temple) and a control sinkhole known as inscriptions, that are separated by large independent catchment areas. The reservoirs were constructed and maintained from the early colonization of Tikal during the Late Preclassic period, ~ 2500 yr. B.P., until the abandonment of the city ~ 1100 yr. B.P.^8,11^. With the exception of the Corriental reservoir (Fig. 1C, Section S2), all excavated reservoirs have stratigraphic discontinuities from dredging during ancient Maya times^7^.

**Figure 2:**
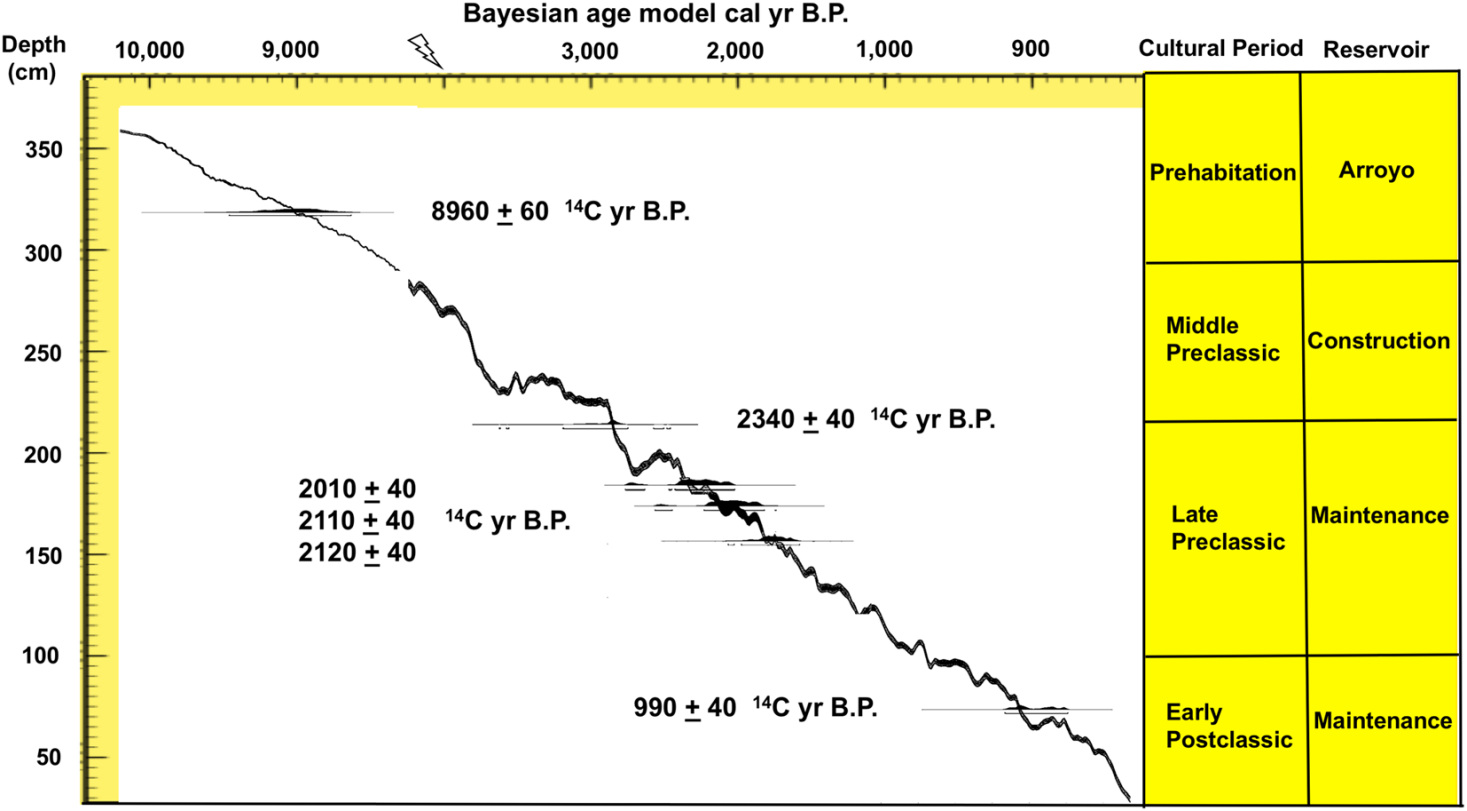
Bayesian age model of radiocarbon ages from OXCAL 4.3. Calibrated radiocarbon ages are plotted by depths below surface, cultural periods, and anthropogenic modifications of the Corriental Reservoir. Kenneth Barnett Tankersley used Microsoft PowerPoint for Mac Version 16.41 (www.microsoft.com) to create this figure.

The ages of the reservoirs and sinkhole sediments were determined by AMS radiocarbon dated charcoal from stratigraphic contexts (Fig. 2, Section S3). The charcoal originated from a continuous rain of carbon from agricultural field clearance, hearth fires and ceramic kilns in surrounding areas during the construction and maintenance of the reservoirs^12,13^. Sixteen AMS radiocarbon ages were obtained from three reservoirs including six samples from Corriental, which date from the Late Preclassic to Early Postclassic cultural periods, ~ 2185–965 cal yr B.P., two ages from Perdido, which date from the Classic cultural period, ~ 2350–1350 cal yr B.P., and four ages from Temple, which date from the Preclassic to the Late Classic cultural periods, ~ 2485–1001 cal yr B.P.^14^. Four additional ages were obtained from the Inscriptions sinkhole, which dates from pre-occupation times to the Early Preclassic period, ~ 13,706–2997 cal yr B.P.^15^. While each reservoir and sinkhole have their own unique depositional history, the timing of sedimentation overlaps between their basins^15,16^.

The volume of sediment in the reservoirs varies depending upon the relative distance to the city center, size and nature of each catchment, and ancient dredging activities. The Temple reservoir was constructed in the city center and had a small catchment area, which was mostly plaster sealed plaza surfaces. Sediment in the main tank of the Temple reservoir is less than a meter-thick^12^. The Corriental and Perdido reservoirs were constructed at lower elevations south of the city center^15^. Perdido received runoff from both a large paved plaza as well as non-paved areas and had a sediment accumulation of about 120 cm above a degraded plaster floor. Corriental received runoff from non-paved surfaces and had an accumulation of sediment of about 250 cm above a well-developed buried pre-Reservoir (Middle Preclassic) clay soil^12^, Section S2.

The mineralogy of the reservoir and sinkhole sediments was determined using X-ray diffraction (XRD) analysis (Section S4). Sediment samples collected at 10 cm intervals from solid-sediment percussion cores, analyzed using XRD, and relative mineral percentages were calculated for each of the samples. XRD analysis demonstrates that all of the sediment samples contain similar quantities of the minerals calcite, smectite, and quartz. Calcite originates from the local Cretaceous-Tertiary limestone bedrock, which forms the karst landscape of Tikal and the South Petén basin^13,14^. Smectite and quartz, however, are volcanogenic in origin. Smectite is a clay mineral derived from the terrestrial alteration of airborne acidic volcanic ash (i.e., glass). Similarly, microcrystalline quartz (~ 50 μm) in the reservoir sediments originated as airborne volcanogenic bipyramidal crystals known as “first quartz”^13^. The ubiquitous co-occurrence of volcanogenic smectite and microcrystalline quartz in the reservoirs and sinkhole sediments suggests that volcanic ash accumulated episodically in the reservoirs throughout the occupation of Tikal^13,14,17^. Pristine, sharp-edged, microcrystalline quartz and zircon crystals in the reservoir sediments also demonstrates that smectite arrived in the reservoirs and sinkholes, and across the southern Maya Lowlands from volcanic ash rather than erosion^17^.

**Figure 3:**
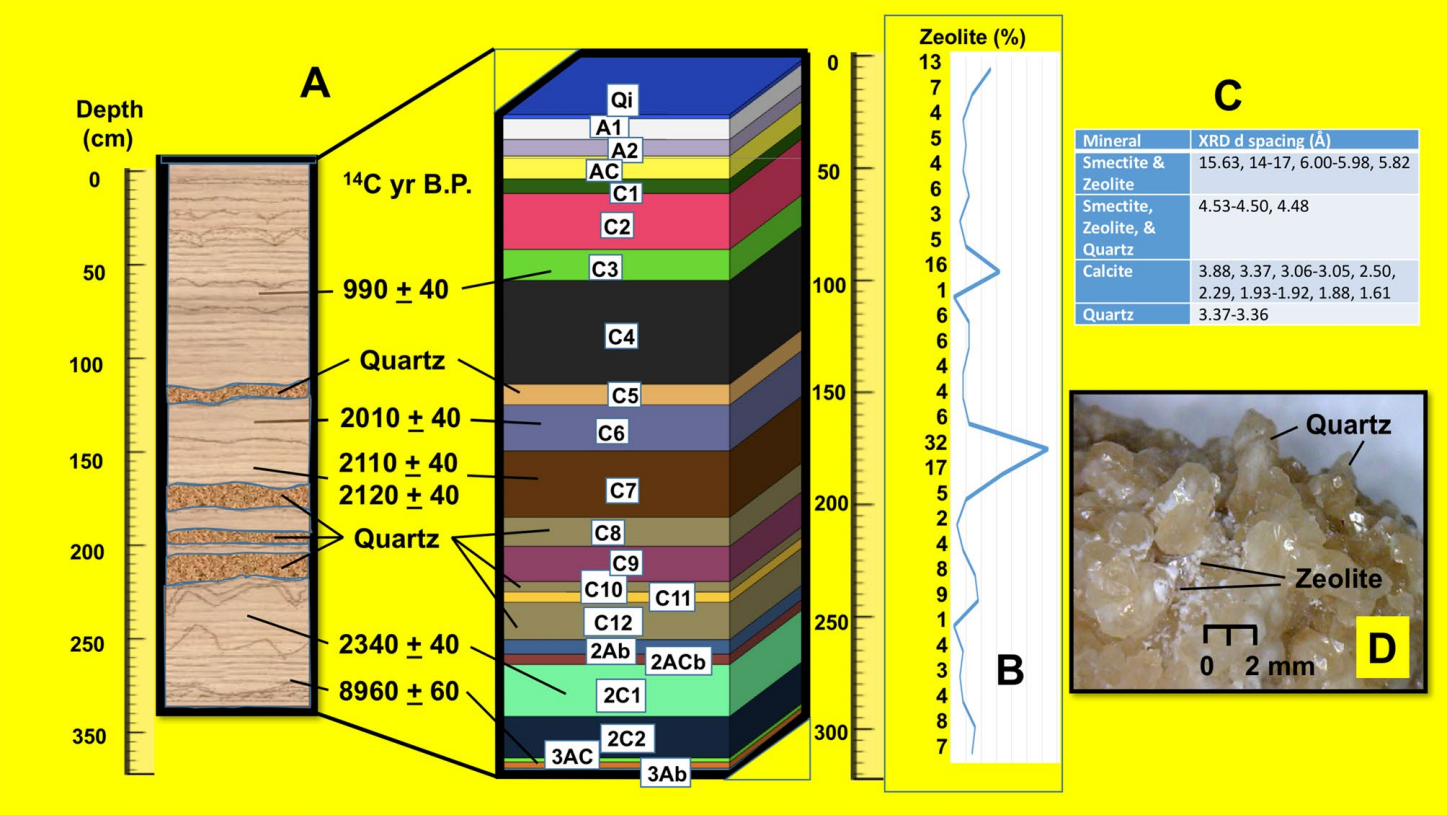
Chronostratigraphy of the Corriental reservoir. (**A**) Soil horizons and sediments of the Corriental reservoir showing the location of anthropogenic quartz and radiocarbon ages yr B.P. (**B**) Relative percent of zeolite by depth. (**C**) Distinctive mineral XRD peaks. (**D**) Photomicrograph of anthropogenic euhedral quartz crystals and zeolite. Kenneth Barnett Tankersley used Microsoft PowerPoint for Mac Version 16.41 (www.microsoft.com) to create this figure.

The mineral zeolite was found solely in the sediments of the Corriental reservoir where it was omnipresent and co-occurs with macro-crystalline (0.5–2.0 mm) euhedral quartz (Fig. 3, Section S4). There are ~ 50 distinct species of zeolites including analcime, clinoptilolite, and mordenite. In Guatemala, analcime occurs as an extensively altered form of jadeite and clinoptilolite and mordenite occur in association with the mineral’s quartz, calcite, and smectite in wet spring settings where volcaniclastic tuffs have altered to zeolites^18–20^. While clinoptilolite and mordenite are not locally available at Tikal, they occur in volcanic rock cavities in western Guatemala where there are active, dormant, and extinct volcanoes^19,21,22^. Clinoptilolite and mordenite have also been discovered in a coarse crystalline Cretaceous-Tertiary tuff exposed northeast of Tikal where clean potable water discharges^23,24^. The co-occurrence of macro-crystalline euhedral quartz, zeolite, and clean drinking water was likely the symbolic connection and empirical basis for the Maya choosing to mine this resource^24,25^.

Zeolite is a non-toxic, three-dimensionally porous, crystalline, hydrated aluminosilicate. Zeolite has adsorbent properties because its three dimensional microcrystalline pore spaces (3–4 Å) create a natural molecular sieve^1^. Consequently, zeolite has the ability to filter out harmful microbes, nitrogenous compounds, and other dispersed insoluble and soluble inorganic and organic toxins from drinking water^26^.

### Corriental and water purification

Corriental is one of the largest reservoirs (~ 58,000,000 L) at Tikal (Fig. 1C, Section S2). Earthenware sherds of water jars of varying size were found in all of Corriental’s strata^11^. Corriental has only minor evidence of chemical pollutants and no evidence of blue-green algal blooms or other pollutants and it is the only excavated reservoir, which was not heavily dredged^11,12,15^. In this regard, Corriental is not only anomalous at Tikal, but throughout the Maya Lowlands (Sections S5–S7). Corriental is also the only reservoir, which has evidence of a zeolite water filtration system.

**Figure 4:**
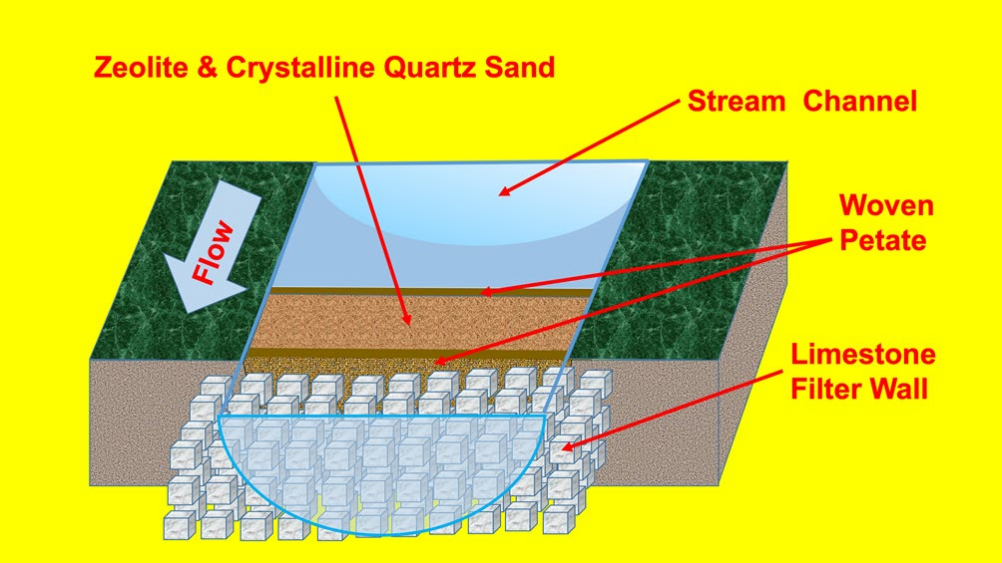
Hypothetical scheme of the ancient water purification system at Tikal. Macro-crystalline quartz crystal sand and zeolite filtration system positioned just upstream of, or within the reservoir ingress. Kenneth Barnett Tankersley used Microsoft PowerPoint for Mac Version 16.41 (www.microsoft.com) to create this figure.

The Corriental water filtration system was composed of clinoptilolite, mordenite, and coarse to very coarse sand-sized euhedral quartz crystals. These zeolites and macro-crystalline quartz are likely derived from a coarsely crystalline Upper Cretaceous tuff stratum exposed along lower margins of deep scarps defining the Bajo de Azúcar located ~ 30 km northeast of Tikal, where it forms an aquifer known locally for its clean water^24^ (Sections S4, S8). The filtration system was likely held behind dry-laid stone walls with the zeolites and macro-crystalline sand-sized quartz crystals further constrained with woven petate (woven reed or palm fiber matting) or other perishable porous material positioned just upstream of, or within the reservoir ingresses, which were periodically ejected into the reservoir during flash floods caused by tropical cyclones^12–14,17^ (Section S9). Evidence for these events can be found in the sequential crystalline quartz lenses in the reservoir sediments (Figs. 3, 4, Sections S2, S4). Because zeolite crystals are much smaller in size (0.1 to < 10 μm), they were more easily water transported and deposited in the reservoir sediments during the functioning of the Corriental reservoir.

While a stand-alone coarse sand-size crystalline quartz filtration system would have been capable of water clarification, it would have had no effect on the removal of harmful microbes or deleterious insoluble or soluble toxins (Section S6). Zeolite was a crucial component of the Corriental water purification system. At Tikal, zeolite occurs in the laminated organic clays of the Corriental reservoir sediments. These strata are indicative of a low energy depositional environment. The clays are interstratified with coarse sand-sized crystalline quartz deposited during periods of high-volume, fast-moving, storm flow (Fig. 3, Section S2). The stratigraphic reoccurrence of embedded macro-crystalline quartz strata suggests that the filtration systems likely had to be replaced following flash-floods^12,13^.

The uppermost stratum in the Corriental reservoir containing zeolite and sand-sized macro-crystalline quartz dates to the Late Classic. This stratum represents the final destruction of the filtration system. In other words, the filtration system was not restored after this event. The subsequent occurrence of anthropogenic mercury (Hg) in the Corriental reservoir likely originated from the weathering of artifactual cinnabar (HgS) in the numerous residential areas and burials within the watershed. Anthropogenically Hg contaminated soil within the Corriental catchment would have in-washed from these areas causing an uptick in Hg^11^ (Section S9). While it is impossible to know why the water filtration system was not rebuilt, it is possible that the Maya no longer had access to the necessary raw materials.

In addition to anthropogenic sources, Hg entering the Corriental reservoir through airborne volcanic activity would not have been filtered out. Volcanogenic Hg can occur in particles as small as 10–20 μm and a single volcanic vent can produce 7000 kg of Hg^27^. Volcanic ashfall on Tikal’s reservoirs originated from strong eruptions of Guatemalan volcanoes and from more distant explosive events from volcanoes in northwestern Chiapas, Mexico such as El Chichón^11,17^. Between March 28 and April 4, 1982, the Maya Lowlands were blanketed by several centimeters of volcanic ash from the El Chichón volcano and concentrations of volcanogenic Hg were enriched by factors of 60 to 20,000^28^. The concentration of airborne volcanogenic Hg in Tikal’s reservoirs would have increased through time during periods of decreased rates of sedimentation.

The Corriental zeolite water purification system was functioning as early as ~ 2185 cal. yr. B.P. (Section S3). The construction of this purification system is ~ 600 years older than the South Asian sand and gravel water filtration described in the Suśrutasaṃhitā (Sushruta Samhita), which dates ~ 1700–1600 years ago^3^. The Corriental system is ~ 1800 years older than Robert Bacon’s sand filtration system developed in 1627 CE and ~ 2155 years older than the first use of zeolite in European water purification systems^3^.

By the Late Preclassic cultural period, the Maya installed a successful and sustainable euhedral quartz and zeolite water purification system at Tikal. This system was critical for survival in a humid tropical environment with unpredictable catastrophic cyclonic and volcanic events, seasonal droughts, and drinking water contaminated by harmful microbes and toxic mineral leachates. Zeolite provided the people of Tikal with safe drinking water for more than 1000 years. It not only represents the oldest Indigenous water filtration system of its kind in the Western Hemisphere, it greatly predates by millennia comparable methods of water purification developed by other cultures in the Old World.

## Conclusions

The ancient Maya city of Tikal adds to our emergent knowledge of the earliest inventions and innovations in water purification. At Tikal, the Maya collected zeolite and euhedral quartz from a coarse crystalline tuff source ~ 30 km northeast of the city between ~ 2185 and 965 cal yr B.P. calendar years ago (cal yr B.P.). The Maya effectively used these natural volcanogenic mineral resources to purify large volumes of drinking water in a tropical forest environment, which was complicated by catastrophic cyclones, volcanic events, droughts, and subsurface drainage. The archaeological record of Tikal includes the oldest known zeolite water purification system that was developed at a time when cultures elsewhere in the world were experimenting with other water purification methods such as boiling, cloth strainers, porous ceramic vessels, and sand sieves.

Tikal was one of the largest Maya cities located in the southern Maya Lowlands, where other Maya centers developed in comparable environmental settings. Together, these sites formed part of the framework for civilization in the southern Maya Lowlands. The reservoir systems of these centers were crucial to their existence, yet water purification systems at these sites remains largely unknown.

The archaeological record of water purification systems is meager in the Western Hemisphere, but it is present at Tikal. The scarcity of archaeological sites with evidence of water decontamination features is mainly due to archaeological visibility and preservation. The Corriental reservoir contains the earliest evidence of a zeolite water purification system. Others may exist, but we don’t know the degree to which Corriental is unique given that there are thousands of ancient Maya reservoirs and less than 50 have been investigated by way of excavation or coring. The earliest water purification systems can only be evaluated and interpreted through interdisciplinary investigations that include chronostratigraphic, mineralogical, and biological analyses, which, if carried out rigorously likely would expose a diverse record of early water decontamination systems not only in the Western Hemisphere, but elsewhere in the ancient world, as well.

## Methods

Sediment samples were obtained from archaeological units and trenches, which were hand excavated under the supervision of members of the University of Cincinnati Tikal Project in 2009 and 2010 (Section S1). Two cm diameter sediment cores were extracted as part of the same project using a hand-operated Environmental Subsoil Probe^7^. Soil horizons and stratigraphic boundaries were defined in the field on the basis of color, texture, structure, and pedogenic features and confirmed in the lab with particle size analysis, magnetic susceptibility, and Munsell soil color charts. The location of all archaeological features, sediment cores, excavation units, and trenches were recorded in the field using a Total station or hand-held GPS by Christopher Carr (Section S10). AMS radiocarbon samples and XRD samples were collected from excavation units, trenches, and solid sediment cores (Sections S1, S3). AMS radiocarbon ages were determined at the Beta Analytic and the Wood’s Hole National Ocean Sciences Accelerator Mass Spectrometry facilities (Section S3). Radiocarbon ages were calibrated and Bayesian analysis was done using OXCAL 4.3. XRD and mineralogical analyses were conducted in the Department of Geology at the University of Cincinnati. Details of the XRD and mineralogical analyses are discussed completely in the Supplementary Materials (Section S4).

## Supplementary information

Supplementary Information.

## Data Availability

All data generated or analyzed during this study are included in this published article (and its Supplementary Information files).
