# Supplementary material for: Zeolite water purification at Tikal, an ancient Maya city in Guatemala
Source: Sci Rep. 2020 Oct 22;10:18021. doi: 10.1038/s41598-020-75023-7 (PMC7582844; doi:10.1038/s41598-020-75023-7)
Supplement: Supplementary file 1 — Supplementary Information. [file 41598_2020_75023_MOESM1_ESM.docx]

**Supplementary Materials for**

**Zeolite water purification at Tikal, an ancient Maya city in Guatemala**

Kenneth Barnett Tankersley^1,2^*†, Nicholas P. Dunning^3^, Christopher Carr^3^, David L. Lentz^4^, Vernon L. Scarborough^1^

^1^Department of Anthropology, University of Cincinnati, Cincinnati, OH 45221, USA.

^2^Department of Geology, University of Cincinnati, Cincinnati, OH 45221, USA.

^3^Department of Geography and GIS, University of Cincinnati, Cincinnati, OH 45221, USA. ^4^Department of Biological Sciences, University of Cincinnati, Cincinnati, OH 45221, USA.

*These authors contributed equally to this work.

†Corresponding author. Email: tankerkh@uc.edu (K.B.T.)

1. **History of archaeological investigations of reservoirs at Tikal**

**
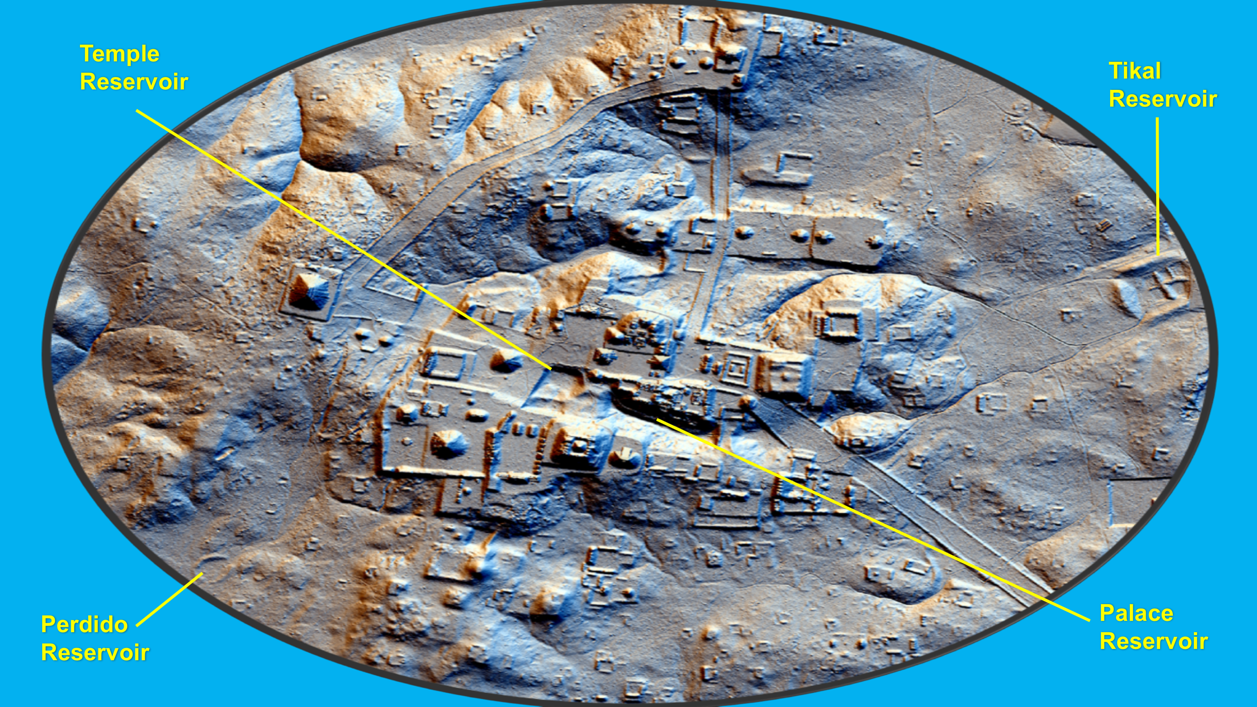
**

**fig. S1. Overview of Tikal.** A PACUNAM lidar-derived hillshade image (S1, S2). Christopher Carr used ESRI ArcGIS 10.3.1 software ([www.esri.com](http://www.esri.com)) to create a GIS layer georeferenced on the hillshades to make the base map using ESRI ArcGIS 10.3.1 software ([www.esri.com](http://www.esri.com)). Kenneth Barnett Tankersley then used Microsoft PowerPoint for Mac Version 16.41 ([www.microsoft.com](http://www.microsoft.com)) to create this figure.

*1.1 20^th^ Century Investigations*

Scientific investigation of the many Maya reservoirs at Tikal began in the 1950s and 1960s as part of the University of Pennsylvania Tikal Project. In fact, Op. 1A, a trench dug for the women’s latrine, was excavated near the “sluiceway” of the Tikal Reservoir (S3). Intriguingly, this excavation exposed a prominent sand lens within its profile. The Penn Project went on to conduct further excavations of various sorts in the Tikal Reservoir, some of these using a bulldozer that was used to deepen the reservoir as the project camp water source.

Excavations were also made in the Palace Reservoir (by Edward Sisson, Christopher Jones, and Peter Harrison), Causeway Reservoir (by Nicholas Hellmuth), Madeira Reservoir (by Edward Sisson and Pat Culbert), Corriental Reservoir (by Donald Callender) and Laguna Verde Aguada on the south national park transect (by Dennis Puleston). Unfortunately, data from these excavations have never been published. In 1968, Donald Callender began to compile information from the various excavations into an unfinished manuscript that was slated to be published in the University of Pennsylvania Museum Tikal monograph series. The partial manuscript and miscellaneous notes were examined in the Museum’s Tikal archive. In 2012, Peter Harrison published a short description of the Penn excavations in the Museum’s journal *Expedition* (S4)*.*

Juan Pedro LaPorte conducted excavations in the Madeira Reservoir sometime in the 1990s, but the resulting data were never published and notes on the investigations could not be located in the archives of the Instituto de Antropología e Historia in Guatemala City (Edwin Román, personal communication, February 29, 2020).

*1.2 21^st^ Century*

Gary Gallopin and Vernon Scarborough used the University of Pennsylvania Tikal Project’s detailed topographic and architectural maps to make a hydrological analysis of the reservoir system within central Tikal (S5, S6, S7). Those analyses formed part of the impetus for the creation of the University of Cincinnati Tikal Project which examined ancient Maya water, forest, and land use around Tikal; excavations were conducted in 2009 and 2010 and laboratory analyses have been ongoing. Excavations were conducted in Palace, Temple (including the “silting tank” or spring pool), Hidden, Perdido, Corriental, and Pital reservoirs in and just south of the site center, as well as the Aguada de Terminos, Aguada Vaca del Monte, and Aguada Elmer in and around the Bajo de Santa Fe several kilometers east of the site center (S8, S9, S10, S11).

As part of the field work, percussion cores were also taken in Corriental. Perdido, and Temple reservoirs and the so-called Inscriptions Reservoir. Cores were also taken with a modified Livingston corer at Aguada de Terminos, Aguada Vaca del Monte, Aguada Pucte, and Tikal Reservoir. Among the findings were the discovery that the feature known as the Silting Tank situated topographically above the Temple Reservoir had been constructed around a natural spring, likely in the Mid- to Late Preclassic period (~ 521-216 BCE). The Palace Reservoir included a system for periodic maintenance and water-level adjustment including stacked sluice portals in the Late Classic dam; this dam encased a smaller Early Classic dam. Geochemical assays of sediments from several of Tikal’s reservoirs indicate that the region received episodic volcanic ash fall. Geochemistry and genetic analyses of several reservoirs have revealed that Temple and Palace reservoirs were highly contaminated with toxic Cyanobacteria and mercury as water levels declined during Terminal Classic droughts in the 9^th^ century CE, whereas Corriental, Perdido, and Terminos reservoirs remained less affected.

**2. Corriental reservoir sediments**

*
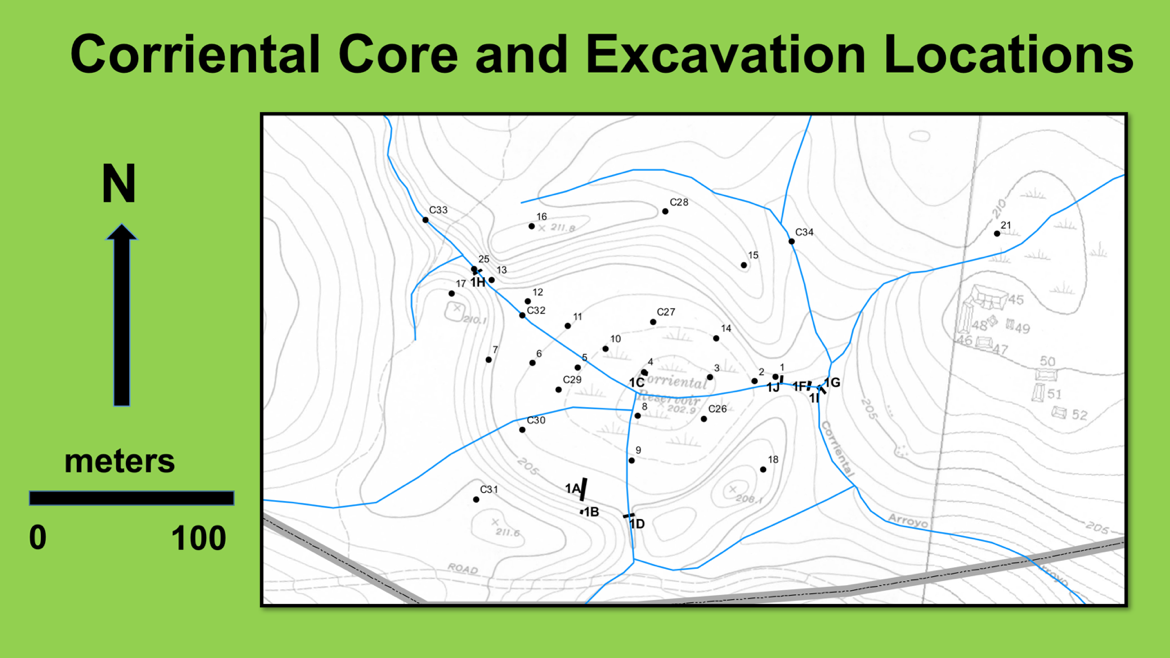
*

**fig. S2. Locations of solid sediment cores and excavation units at the Corriental reservoir.** This map was produced by Christopher Carr from hard copies of the University of Pennsylvania maps of Tikal to an electronic format for use in ESRI ArcGIS 10.3.1 software ([www.esri.com](http://www.esri.com)). Kenneth Barnett Tankersley then used Microsoft PowerPoint for Mac Version 16.41 ([www.microsoft.com](http://www.microsoft.com)) to create this figure.

*2.1 Excavation trench 1C*.

Op 1C was 1 x 1.5 m trench excavated near the center point of the Corriental Reservoir. The pit reached a final depth of 3.15 m, at which point weathered limestone bedrock was encountered. Ceramics recovered in this operation were analyzed by Dr. Pat Culbert at Tikal. Strata within the lower portions of the trench indicate that the Corriental was probably constructed within a small, localized natural depression (micro bajo). The lowermost strata in the pit (3Ab and 3AC horizons) are a highly compacted, skeletal soil. Based on similarities to other deeply buried soils in small bajos elsewhere in the northeast Peten and northwest Belize, this soil is likely of late Pleistocene age (~11,000 – 13,000 BP) (S11, S12).

With the onset of wetter conditions in the Holocene period, the micro depression filled in with sediment eroded from upslope areas (2C1 and 2C2 horizons) on which a new soil surface (2Ab and 2AC horizons) gradually developed. Radiocarbon dating of organic matter with the 2Ab horizon place the age of this soil surface at approximately 760 – 400 BCE (Middle Preclassic). However, this date is based on accumulated organic matter within the soil (organic matter which accumulates over hundreds of years), hence the soil surface was actually likely last exposed sometime in the late Preclassic. This soil is a Vertisol typical of small seasonally wet/dry bajos in the Peten. Four highly weathered sherds were recovered from this level, but were non-diagnostic.

At some point within the next 500 years, drainage within the depression appears to have been substantially modified and sediment began to accumulate more rapidly. This modification likely corresponds to the initial construction of the Corriental Reservoir: probably accomplished by a combination of quarrying (widening the natural depression), up building of the encircling berm, and diversion of inflowing streams. Subsequently, the floor of the reservoir began to aggrade (fill with sediment) over time. Alternating strata of laminated carbonate sand and small, rounded gravel and organic clay are found between 65 and 253 cm depth within the trench (C3 to C12 horizons). The laminated or stratified nature of the sandy deposits indicates that these sands were deposited by fluvial (running water) processes.

One explanation is that sand was used to filter water entering the reservoir and that sand from the filters was occasionally flushed into the reservoir proper by storm-related flooding. On the other hand, the organic clay layers are typical of still water deposits and likely accumulated very slowly while the reservoir was in active use. There are no obvious signs of dredging within the sediments revealed in Op 1C.

One probable stratigraphic disjunction occurs early in the Op 1C sequence, namely between the 2Ab and C12 horizons. The 2Ab horizon, the topsoil of the Middle Preclassic Vertisol appears to be somewhat truncated, likely scoured by a major flood event that removed portions of this soil horizon and perhaps some amount of overlying reservoir sediment. That flood event also likely dropped the coarse sand and large, flat cobble that form the C12 horizon. We interpret this sequence as the product of a major storm event that blew out the first of the sand filters, including, including the stone filter wall, probably late in the Late Preclassic period or early in the Early Classic period (ca. 0 – 300 AD). Prior to the Late Preclassic period Tikal was a small community that likely did not have the economic or political wherewithal to procure sand from the Bajo de Azúcar, but grew in size and power in the Late Preclassic and continuing into the Classic period.

Ceramic sherds were recovered in the C3, C5, C8, C10, and C12 horizons (i.e., principally in the sandy strata. Small and large water jar forms predominate in all strata. C12, the deepest alluvial stratum, included identifiable Early Classic types. C10 had no diagnostic sherds. C8 contained a mix of Early and Late Classic types. C5 included only Late Classic types. C3 had no diagnostic sherds. Charcoal within the C3 horizon (65 cm) produced an AMS radiocarbon date of CE 1010 – 1170, suggesting that the reservoir may have continued to be in use to some extent as late as the Early Postclassic.

Subsequently, there is no evidence of continued use of the reservoir, though it has naturally continued to seasonally collect water. The modern soil that has developed within the reservoir (Oi through C2 horizons) is a Terric Fibrist, an organic soil with mineral subsoil typical of regional depressions which remain partially moist year-round.

The Corriental Reservoir, on the southern flank of central Tikal, appears to have been constructed sometime towards the end of the Late Preclassic or very early in the Early Classic period. It was constructed by widening a pre-existing natural small depression by quarrying, mounding earth to form an encircling berm, and diverting water flow from a local stream. Thick sediment deposits within the reservoir included alternating deposits of stratified carbonate sands and organic clays. The clays indicate periods of stability during which clay and organic matters gradually settled onto the reservoir floor. The sandy strata are indicative of running water, perhaps deposited during higher-energy storm runoff events. The origin of the sand is unclear, but it may have been used to filter water as it entered the reservoir, and then was occasionally flushed into the reservoir proper during flooding. Ceramics recovered from within the reservoir sediments were generally very weathered, but contained a mixture of Early Classic and Late Classic types. Most notable was the presence of quantities of huge jar fragments.

**table S1. Description of soils exposed in excavation trench 1C in the center of Corriental reservoir.**

| **Horizon** | **Depth**  **(cm)** | **Munsell**  **Soil Color** | **Soil Description** |
| --- | --- | --- | --- |
| Oi | 0-2 | Very dark gray (5YR3/1) | Fibric organic matter |
| A1 | 2-12 | Very dark brown (7.5YR2.5/2) | Organic clay; large, hard crumbs |
| A2 | 12-20 | Black (10YR2/1) | Organic clay; large, subangular blocks |
| ACss | 20-31 | Dark gray (10YR3/1) | Clay; massive; faint slickensides |
| C1ss | 31-38 | Gray (5Y5/1) | Sandy clay (± 10% coarse limestone sand); massive; faint slickensides |
| C2ss | 38-65 | Gray (5Y6/1) | Clay; massive; faint slickensides |
| C3 | 65-80 | Gray (5Y5/1) | Clay; massive; scattered sherds and Pomacaea shells; charcoal (calibrated age AD 1010–1170); 4 sherds (non- diagnostic) |
| C4ss | 80-130 | Dark gray (5Y4/1) | Organic clay; massive; faint slickensides |
| C5 | 130-140 | Light olive gray (5Y6/2) | Stratified coarse sand and pea gravel in clay matrix; 28 sherds (Lot 5: large and small jars; Late Classic artifact diagnostics) |
| C6ss | 140-162 | Gray (5Y5/1) | Clay; massive; slickensides |
| C7 | 162-194 | Dark gray (5Y4/1) | Organic clay; massive |
| C8 | 194-208 | Very pale brown (10YR7/3) | Stratified coarse sand and gravel in clay matrix; 69 sherds (Lot 7: large and small jars; both Early and Late Classic types) |
| C9 | 208-225 | Dark gray (5Y4/1) | Organic clay; massive |
| C10 | 225-230 | Very pale brown (10YR7/3) | Stratified coarse sand and gravel in clay matrix; scattered small cobbles; 19 sherds (Lot 8: 1 large jar; no diagnostics) |
| C11ss | 230-235 | Dark gray (5Y4/1) | Organic clay; massive; slickensides |
| C12 | 235-253 | Very pale brown (10YR7/3) | Stratified coarse sand and gravel in clay matrix; several large rocks; 35 sherds (Lot 9: many large and small jars; some probable Early Classic types) |
| 2Abss | 253-260 | Dark gray (2.5Y4/1) | Clay; massive; slickensides; 4 sherds (Lot 10: non-diagnostic); humate calibrated radiocarbon date: BCE 760 – 400 |
| 2ACbss | 260-265 | Gray (2.5Y5/1) | Clay; massive; slickensides |
| 2C1bss | 265-290 | Light gray (Gley7/N) | Clay; massive; slickensides; Mn oxide nodules; cherty pebbles |
| 2C2bss | 290-310 | Gray (Gley6/N) | Clay; massive; slickensides; cherty pebbles |
| 3Abss | 310-312 | Black (Gley2.5/N) | Organic clay; massive |
| 3ACbss | 312-315 | Light greenish gray (Gley10Y8/2) | Clay and weathered cherty limestone |
| R | 315+ |  | Weathered cherty limestone bedrock |

**table S2. Chemistry of soils exposed in excavation trench 1C in the center of Corriental reservoir performed by Spectrum Analytic, Washington Courthouse, Ohio.**

| **Soil**  **Horizon^1^** | **Depth**  **(cm)** | **pH** | **OM**  **(%)** | **Ca**  **(ppm)** | **P^2^**  **(ppm)** | **Mg**  **(ppm)** | **K**  **(ppm)** | **S^3^**  **(ppm)** |
| --- | --- | --- | --- | --- | --- | --- | --- | --- |
| A1 | 10 | 7.1 | 4.5 | 13,662 | 52 | 133 | 59 | 81 |
| C2 | 50 | 7.0 | 1.9 | 13,877 | 38 | 150 | 66 | 65 |
| C3 | 70 | 7.2 | 2.0 | 14,316 | 289 | 167 | 64 | 69 |
| C4 | 100 | 7.5 | 2.6 | 15,014 | 237 | 165 | 60 | 70 |
| C5 | 135 | 7.8 | 0.8 | 22,474 | 299 | 173 | 67 | 68 |
| C6 | 150 | 7.6 | 1.8 | 16,269 | 394 | 190 | 69 | 77 |
| C7 | 180 | 8.0 | 2.2 | 18,406 | 363 | 182 | 82 | 74 |
| C8 | 200 | 7.9 | 1.0 | 21,435 | 280 | 184 | 65 | 56 |
| C9 | 220 | 7.8 | 2.5 | 19,270 | 370 | 285 | 108 | 287 |
| C10 | 228 | 7.9 | 0.7 | 21,025 | 282 | 191 | 71 | 142 |
| C11 | 233 | 7.7 | 2.9 | 18,855 | 322 | 292 | 91 | 201 |
| C12 | 240 | 8.1 | 1.0 | 23,984 | 176 | 123 | 64 | 58 |
| 2Ab | 255 | 7.8 | 3.1 | 16,098 | 211 | 304 | 36 | 197 |
| 2AC | 275 | 7.9 | 2.5 | 15,800 | 190 | 361 | 30 | 203 |
| 3Ab | 310 | 7.7 | 3.8 | 12,149 | 41 | 299 | 35 | 308 |

1. O, A2, AC, and C1 horizons were not tested.
2. P enrichment is highest in ponded, organic clay sediments that likely accumulated slowly episodically within the reservoir.
3. High S levels and redox features in 2Ab and 3Ab horizons indicate that these buried soils were hydromorphic (formed in a swampy environment).

*2.2 Particle size analysis.*

Sediment textures in the Corriental reservoir were determined on the basis of particle size analysis, a two-­dimensional estimate of particle size distributions (i.e., the percent of particles of different sizes). In addition to information about the physical composition of the sediment, particle size analysis provided important data concerning the depositional energy and environment of the Corriental reservoir through time.

Particle size analysis was accomplished using a stack of 9 brass sieves (> 2360, 2000, 1700, 1180, 850, 600, 420, 75, and < 75 µm respectively), which conformed to the International Organization for Standardization Specification (IOSS). Sediment samples were subjected to dry vibratory sieving using an electronic sieve shaker with an adjustable continuous amplitude and an electromagnetic drive. Sediment samples were placed on the surface of the sieve stack, which was then sealed and vibrated until particles were no longer passing through the sieves. Each sieve was then separated and the contents weighed. The weight proportion of each of the 9 sieves was calculated as a percentage of the mass of the whole soil sample.


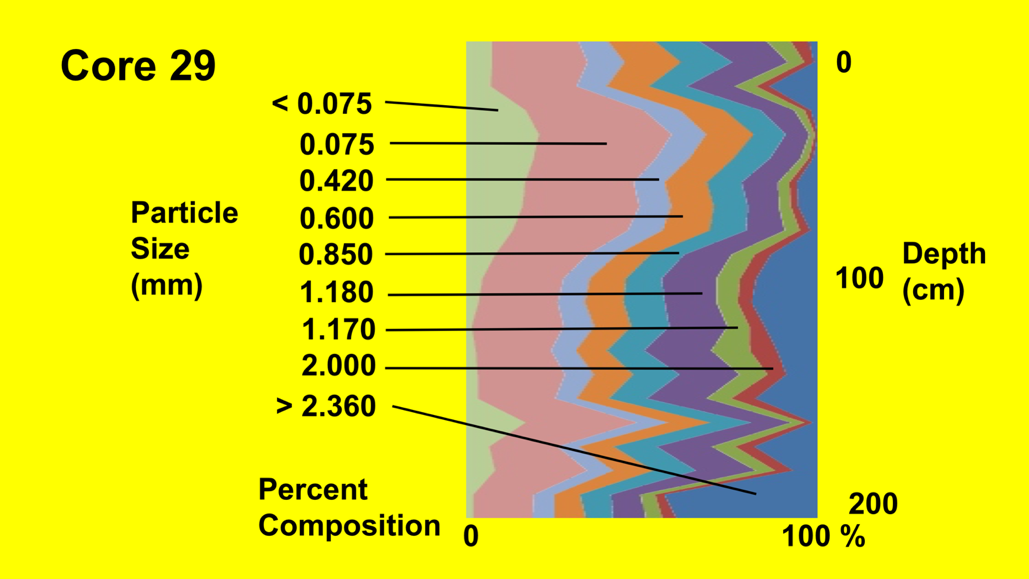


**fig. S3 Percent composition of sediment samples extracted from Corriental Core 29.** Kenneth Barnett Tankersley used Microsoft PowerPoint for Mac Version 16.41 ([www.microsoft.com](http://www.microsoft.com)) to create this figure.

**table S.3 Munsell color and particle size of solid sediment core samples from the Corriental reservoir.**

| **Depth**  **(cm)** | **Munsell Color** | **>2360**  **(µm)** | **2000 (µm)** | **1700 (µm)** | **1180 (µm)** | **850 (µm)** | **600 (µm)** | **420 (µm)** | **75 (µm)** | **<75 (µm)** |
| --- | --- | --- | --- | --- | --- | --- | --- | --- | --- | --- |
| **Core 3** | | | | | | | | | | |
| 0-10 | 2.5Y2.5/1 | 6.52 | 2.71 | 4.87 | 10.9 | 7.79 | 6.04 | 2.98 | 4.45 | 0.00 |
| 10-20 | 2.5Y2.5/1 | 1.48 | 1.42 | 2.73 | 7.57 | 7.00 | 6.47 | 3.49 | 5.90 | 0.00 |
| 20-30 | 2.5Y4/1 | 0.59 | 0.72 | 2.03 | 6.58 | 8.04 | 8.32 | 5.53 | 10.8 | 0.00 |
| 30-40 | 2.5Y4/1 | 6.20 | 3.29 | 4.69 | 8.7 | 6.92 | 6.07 | 3.98 | 11.14 | 0.00 |
| 40-50 | 2.5Y4/1 | 3.17 | 3.65 | 4.76 | 9.55 | 7.86 | 6.75 | 6.15 | 11.45 | 0.00 |
| 50-60 | 2.5Y4/1 | 10.45 | 4.82 | 6.04 | 9.52 | 6.31 | 4.49 | 2.53 | 4.66 | 0.00 |
| 60-70 | 2.5Y4/1 | 18.28 | 6.70 | 5.81 | 6.50 | 4.14 | 2.53 | 1.60 | 3.25 | 0.00 |
| 70-80 | 2.5Y5/1 | 19.37 | 4.78 | 4.80 | 7.14 | 3.79 | 2.92 | 1.70 | 3.04 | 0.00 |
| 80-90 | 2.5Y4/1 | 14.06 | 5.01 | 5.73 | 8.23 | 4.62 | 3.67 | 2.28 | 4.15 | 0.00 |
| 90-100 | 2.5Y4/1 | 23.36 | 6.30 | 4.04 | 6.15 | 3.44 | 2.55 | 1.92 | 3.82 | 0.00 |
| 100-110 | 2.5Y3/1 | 16.12 | 3.14 | 3.60 | 5.58 | 3.99 | 4.65 | 2.29 | 7.29 | 0.78 |
| 110-120 | 2.5Y3/1 | 12.09 | 3.60 | 3.17 | 7.18 | 4.18 | 3.58 | 2.22 | 5.94 | 1.37 |
| 120-130 | 2.5Y4/1 | 12.92 | 4.74 | 4.46 | 7.55 | 4.32 | 4.10 | 2.73 | 6.94 | 1.90 |
| **Core 4** | | | | | | | | | | |
| 0-10 | 10YR2/2  10YR4/3  2.5Y7/4 | 0.02 | 0.01 | 0.15 | 0.77 | 1.40 | 2.00 | 1.53 | 5.33 | 1.28 |
| 10-20 | 10YR2/1  10YR6/6 | 0.10 | 0.13 | 0.31 | 1.71 | 2.58 | 3.83 | 2.97 | 8.47 | 2.19 |
| 20-30 | 10Y4/1 | 1.65 | 1.35 | 1.42 | 5.11 | 4.98 | 5.83 | 4.54 | 14.86 | 7.84 |
| 30-36 | 10YR6/1  10YR8/2 | 2.39 | 0.43 | 0.54 | 2.11 | 2.53 | 3.31 | 2.75 | 9.22 | 5.15 |
| 40-50 | 10YR6/1  10YR5/2  10YR2/1  10YR2/2 | 0.16 | 0.33 | 0.67 | 2.90 | 4.05 | 5.06 | 4.45 | 14.76 | 8.66 |
| 50-60 | 10YR6/1  10YR5/1  10YR8/2 | 0.65 | 0.41 | 0.78 | 3.55 | 4.62 | 5.70 | 4.58 | 15.75 | 8.10 |
| 60-70 | 10YR6/1  10YR5/1  10YR8/2 | 0.75 | 0.61 | 0.77 | 3.62 | 3.83 | 5.71 | 4.26 | 15.73 | 6.67 |
| 70-80 | 10YR6/1  10YR7/1  10YR8/2 | 0.42 | 0.19 | 0.31 | 1.71 | 3.05 | 5.79 | 5.44 | 20.51 | 11.22 |
| 80-90 | 10YR6/1  10YR7/1  10YR8/1  10YR5/6 | 7.20 | 0.89 | 1.05 | 4.01 | 4.61 | 6.55 | 4.63 | 17.93 | 3.11 |
| 90-100 | 10YR6/1  10YR7/1  10YR8/1 | 0.85 | 1.13 | 1.35 | 4.46 | 5.10 | 7.19 | 5.84 | 19.24 | 3.59 |
| 100-110 | 10YR6/1  10YR5/1  10YR8/1  10YR7/1 | 1.83 | 1.63 | 1.57 | 4.6 | 5.08 | 5.65 | 4.88 | 21.17 | 1.73 |
| 110-123 | 10YR5/1  10YR6/1  10YR7/1  10YR8/1 | 2.13 | 1.30 | 1.24 | 4.86 | 6.11 | 8.06 | 6.96 | 25.58 | 2.22 |
| **Core 8** | | | | | | | | | | |
| 0-10 | GreY1 2.5/N | 0.62 | 0.20 | 0.22 | 0.22 | 0.13 | 0.12 | 0.11 | 0.17 | 0.00 |
| 10-20 | 7.5YR5/1 | 0.12 | 0.16 | 0.13 | 0.38 | 0.28 | 0.21 | 0.16 | 0.29 | 0.00 |
| 20-30 | 7.5YR5/1 | 0.75 | 0.27 | 0.31 | 0.23 | 0.16 | 0.08 | 0.04 | 0.06 | 0.00 |
| 30-40 | 7.5YR5/1 | 0.74 | 0.14 | 0.13 | 0.24 | 0.21 | 0.16 | 0.13 | 0.3 | 0.00 |
| 40-50 | 7.5YR5/1 | 0.32 | 0.32 | 0.21 | 0.32 | 0.17 | 0.13 | 0.12 | 0.25 | 0.00 |
| 50-60 | 7.5YR5/1 | 0.15 | 0.18 | 0.13 | 0.32 | 0.25 | 0.21 | 0.17 | 0.45 | 0.00 |
| 60-70 | 7.5YR5/1 | 0.30 | 0.11 | 0.23 | 0.33 | 0.23 | 0.20 | 0.12 | 0.22 | 0.00 |
| 70-80 | 7.5YR5/1 | 0.17 | 0.24 | 0.19 | 0.36 | 0.25 | 0.20 | 0.15 | 0.25 | 0.00 |
| 80-90 | 7.5YR2.5/1 | 5.70 | 7.20 | 3.80 | 3.72 | 1.82 | 3.12 | 1.73 | 4.78 | 0.00 |
| 90-100 | 2.5Y4/1 | 2.93 | 2.53 | 3.76 | 6.89 | 4.95 | 4.21 | 2.76 | 6.47 | 0.00 |
| 100-110 | 2.5Y4/1 | 4.54 | 2.48 | 3.09 | 5.71 | 4.40 | 4.09 | 2.83 | 8.16 | 0.00 |
| 110-120 | 2.5Y4/1 | 3.01 | 2.18 | 2.31 | 4.63 | 4.54 | 4.02 | 2.97 | 7.46 | 0.00 |
| 120-130 | 2.5Y5/1 | 5.97 | 3.68 | 3.60 | 5.56 | 4.07 | 3.21 | 2.20 | 5.06 | 0.00 |
| 130-140 | 2.5Y4/1 | 8.85 | 3.80 | 4.12 | 5.67 | 3.86 | 3.10 | 2.69 | 5.28 | 0.00 |
| 140-150 | 2.5Y4/1 | 1.98 | 1.52 | 2.42 | 3.56 | 3.60 | 3.51 | 2.57 | 6.56 | 0.00 |
| 150-160 | 2.5Y4/1 | 3.44 | 1.56 | 2.31 | 4.72 | 3.93 | 3.91 | 2.82 | 8.38 | 0.00 |
| 160-170 | 7.5YR2.5/1 | 4.45 | 2.84 | 2.78 | 5.88 | 4.01 | 3.16 | 1.84 | 3.58 | 0.00 |
| 170-180 | 7.5YR2.5/1 | 4.61 | 1.40 | 0.88 | 1.03 | 0.52 | 0.32 | 0.16 | 0.25 | 0.00 |
| 180-190 | 7.5YR2.5/1 | 3.08 | 1.28 | 2.91 | 0.00 | 0.98 | 0.71 | 0.41 | 0.89 | 0.00 |
| 190-200 | 7.5YR2.5/1 | 7.18 | 3.12 | 0.00 | 9.05 | 3.62 | 2.89 | 1.84 | 4.23 | 0.00 |
| 200-210 | 7.5YR2.5/1 | 7.24 | 2.03 | 1.99 | 3.14 | 1.92 | 2.03 | 1.18 | 2.99 | 0.00 |
| 210-220 | 7.5YR5/1 | 7.71 | 1.81 | 2.29 | 3.77 | 3.03 | 2.46 | 1.60 | 3.81 | 0.00 |
| 220-230 | 7.5YR5/1 | 15.24 | 2.34 | 2.53 | 3.16 | 2.16 | 2.65 | 1.62 | 5.34 | 0.00 |
| 230-240 | 2.5Y2.5/1 | 21.65 | 1.51 | 1.34 | 2.27 | 1.95 | 1.96 | 1.58 | 5.84 | 0.00 |
| 240-250 | 2.5Y2.5/1 | 13.32 | 1.50 | 1.23 | 2.52 | 2.16 | 2.86 | 2.44 | 8.40 | 0.00 |
| 250-260 | 2.5Y2.5/1 | 16.03 | 0.68 | 0.65 | 1.22 | 1.11 | 1.39 | 1.26 | 4.70 | 0.00 |
| 260-270 | 2.5Y2.5/1 | 17.00 | 1.71 | 1.66 | 2.91 | 2.53 | 2.78 | 2.31 | 6.84 | 0.00 |
| 270-280 | 2.5Y5/1 | 11.90 | 2.16 | 2.29 | 3.99 | 3.35 | 3.09 | 3.04 | 9.04 | 0.00 |
| 280-290 | 2.5Y7/1  2.5Y7/6 | 37.26 | 1.20 | 1.21 | 1.44 | 0.74 | 0.51 | 0.48 | 0.99 | 0.00 |
| 290-300 | 2.5Y7/1  2.5Y7/6 | 27.53 | 1.62 | 1.27 | 1.38 | 0.55 | 0.49 | 0.38 | 0.87 | 0.00 |
| 300-310 | 2.5Y7/1  2.5Y7/8 | 21.5 | 3.12 | 2.39 | 2.87 | 1.07 | 0.81 | 0.49 | 1.16 | 0.00 |
| 310-320 | 2.5Y7/1  2.5Y7/6 | 16.9 | 4.52 | 4.08 | 5.65 | 2.78 | 2.18 | 1.34 | 3.21 | 0.00 |
| **Core 10** | | | | | | | | | | |
| 0-10 | 10YR2/1  10YR2/2  2.5Y3/3 | 1.62 | 0.64 | 0.79 | 3.15 | 3.23 | 3.84 | 3.00 | 10.81 | 5.41 |
| 10-20 | 10YR2/1  10YR2/2  2.5Y3/1  2.5Y4/1 | 1.93 | 0.36 | 0.71 | 3.49 | 4.63 | 5.79 | 4.39 | 16.58 | 10.26 |
| 20-30 | 2.5Y3/1  2.5Y4/1  2.5Y4/2  2.5Y8/3 | 1.52 | 0.88 | 1.18 | 4.94 | 5.11 | 6.02 | 5.13 | 16.48 | 8.48 |
| 30-40 | 2.5Y4/1  2.Y8/3  2.5Y8/2  2.5Y8/1 | 21.88 | 1.14 | 1.51 | 3.41 | 2.85 | 2.85 | 2.28 | 8.22 | 2.80 |
| 40-50 | 2.5Y4/1  2.5Y8/2  2.5Y8/3  2.5Y8/1 | 16.09 | 1.14 | 1.34 | 3.18 | 2.79 | 3.31 | 2.65 | 8.31 | 3.41 |
| 50-60 | 2.5Y4/1  2.5Y4/2  2.5Y8/2  2.5Y8/1 | 2.66 | 0.51 | 0.76 | 3.58 | 4.71 | 6.06 | 4.91 | 17.97 | 10.42 |
| 60-70 | 2.5Y4/1  2.5Y8/1  2.5Y8/2 | 7.20 | 0.77 | 1.12 | 4.38 | 4.31 | 5.24 | 4.32 | 15.70 | 8.13 |
| 70-80 | 2.5Y6/1  2.5Y8/2  10YR4/1  10YR6/6  2.5Y7/1 | 11.15 | 1.65 | 1.16 | 3.47 | 3.23 | 3.26 | 2.44 | 9.20 | 0.65 |
| 80-90 | 10YR6/1  10YR8/1  10YR 8/4 | 18.73 | 2.42 | 2.19 | 5.46 | 4.63 | 4.16 | 3.09 | 11.47 | 0.57 |
| 90-100 | 10YR4/1  10YR6/2  10YR7/6 | 17.81 | 2.89 | 2.66 | 6.02 | 5.11 | 4.48 | 3.34 | 11.68 | 0.18 |
| 100-110 | 10YR3/1  10YR6/1  10YR5/6  10YR 8/1 | 21.57 | 2.75 | 1.96 | 3.73 | 2.85 | 2.75 | 2.39 | 10.59 | 0.18 |
| 110-120 | 10YR4/1  10YR8/3 | 22.65 | 1.96 | 1.79 | 4.39 | 2.79 | 3.38 | 2.4 | 12.08 | 0.31 |
| 120-130 | 10YR4/1  10YR7/3  10YR6/8  10YR3/3 | 8.20 | 1.30 | 1.33 | 3.75 | 4.71 | 6.54 | 4.79 | 21.74 | 1.83 |
| 130-140 | 10YR8/1  10YR7/6  10YR3/4 | 10.92 | 1.56 | 1.35 | 2.93 | 4.31 | 4.70 | 3.80 | 12.67 | 2.08 |
| 144-150 | 2.5Y6/1  2.5Y8/2 | 1.13 | 0.20 | 0.23 | 1.58 | 2.93 | 2.24 | 1.63 | 5.71 | 2.11 |
| 150-160 | 10YR2/1  10YR8/1  10YR6/1  5YR5/8  10YR7/4 | 7.53 | 1.14 | 1.04 | 3.08 | 4.06 | 4.95 | 3.93 | 16.23 | 0.48 |
| 160-170 | 10YR5/1  10YR6/8  10YR8/2  10YR7/8 | 18.12 | 1.62 | 1.5 | 3.91 | 4.21 | 3.98 | 3.1 | 12.8 | 0.70 |
| 170-180 | 10YR4/1  10YR8/6  10YR8/2  10YR8/4 | 8.80 | 1.80 | 1.78 | 4.50 | 2.69 | 4.95 | 5.78 | 12.97 | 0.36 |
| 180-190 | 10YR4/1  10YR8/6  10YR8/2 | 4.38 | 1.82 | 2.08 | 5.00 | 2.79 | 5.81 | 5.07 | 16.79 | 6.20 |
| 190-200 | 10YR4/1  10YR6/8  10YR8/1 | 2.39 | 1.29 | 1.27 | 4.37 | 4.41 | 4.32 | 3.10 | 14.76 | 0.89 |
| 200-205 | 10YR5/1  10YR7/6  10YR8/4 | 4.83 | 0.38 | 0.47 | 1.26 | 3.81 | 1.27 | 1.11 | 4.91 | 0.12 |
| **Core 11** | | | | | | | | | | |
| 0-10 | 2.5Y2.5/1 | 0.08 | 0.36 | 0.39 | 1.91 | 2.58 | 3.76 | 3.15 | 12.4 | 6.51 |
| 10-20 | 2.5Y2.5/1  2.5Y8/1 | 12.67 | 0.18 | 0.38 | 1.83 | 2.98 | 4.62 | 3.78 | 14.83 | 9.54 |
| 20-30 | 10YR5/1  10YR8/1 | 2.88 | 0.27 | 0.29 | 2.22 | 3.60 | 5.6 | 4.50 | 15.96 | 9.39 |
| 30-40 | 2.5Y7/1  2.5Y8/1  10YR8/6 | 8.99 | 0.18 | 0.29 | 1.52 | 2.96 | 4.79 | 4.86 | 17.3 | 10.95 |
| 40-50 | 10YR5/1  2.5Y8/3  10YR8/1 | 0.3 | 0.12 | 0.29 | 2.33 | 4.07 | 6.25 | 6.09 | 21.65 | 12.82 |
| 50-60 | 2.5Y5/1  2.5Y8/1 | 13.70 | 0.37 | 0.44 | 1.90 | 2.77 | 4.72 | 4.87 | 18.99 | 12.16 |
| 60-70 | 2.5Y5/1  2.5Y8/1  2.5Y3/1  2.5Y8/3 | 5.01 | 0.41 | 0.54 | 1.29 | 1.41 | 1.45 | 1.25 | 4.89 | 0.07 |
| 70-80 | 2.5Y3/1  2.5Y4/1  2.5Y8/3  2.5Y8/1 | 3.12 | 0.14 | 0.22 | 1.68 | 2.97 | 5.93 | 5.90 | 23.32 | 13.86 |
| 80-90 | 10YR6/1  10YR6/2  10YR7/4  10YR4/6  10YR8/1 | 6.30 | 0.39 | 0.73 | 2.87 | 4.35 | 6.42 | 5.15 | 18.63 | 13.02 |
| 90-100 | 2.5Y5/2  2.5Y8/3  2.5Y8/1  2.5YR7/8 | 8.06 | 0.93 | 1.25 | 4.41 | 4.88 | 6.45 | 5.25 | 19.12 | 10.46 |
| 100-110 | 2.5Y4/1  2.5Y7/3  2.5Y8/3  2.5Y8/2 | 5.69 | 0.77 | 1.03 | 3.86 | 4.33 | 6.17 | 4.68 | 18.33 | 7.67 |
| 110-120 | 2.5Y4/1  2.5Y5/3  2.5Y8/1 2.5Y8/2 | 4.37 | 0.97 | 1.79 | 5.25 | 5.57 | 6.91 | 5.21 | 18.16 | 8.76 |
| 120-130 | 2.5Y5/1 2.5Y8/3 2.5Y8/2 2.5YR7/8 | 8.92 | 1.48 | 1.65 | 5.08 | 4.76 | 4.96 | 4.40 | 16.86 | 7.64 |
| 130-140 | 2.5Y2.5/1 2.5Y5/1 2.5Y5/4 2.5Y8/3 2.5Y8/1 | 10.42 | 1.01 | 1.11 | 3.47 | 3.83 | 5.12 | 5.23 | 19.82 | 7.87 |
| 140-145 | 2.5Y2.5/1 2.5Y3/1  2.5Y8/4 2.5Y8/2 2.5Y8/1 | 3.25 | 0.17 | 0.26 | 0.92 | 1.74 | 3.50 | 3.28 | 11.25 | 5.38 |
| 152-160 | 2.5Y4/1 2.5Y7/3 2.5Y8/2 2.5Y8/1 | 7.93 | 0.58 | 0.87 | 2.82 | 2.80 | 3.13 | 2.57 | 9.68 | 4.15 |
| 160-170 | 2.5Y5/1 2.5Y4/1  2.5Y8/2 2.5Y8/1 | 8.61 | 1.59 | 2.19 | 6.02 | 5.44 | 6.05 | 4.81 | 15.55 | 7.39 |
| 170-178 | 2.5Y5/1 2.5Y4/1 2.5Y8/1 | 4.72 | 0.88 | 1.18 | 3.41 | 3.06 | 3.39 | 2.57 | 8.77 | 4.76 |
| **Core 12** | | | | | | | | | | |
| 0-10 | 10YR2/2 | 0.08 | 0.07 | 0.19 | 2.22 | 3.40 | 4.02 | 3 | 9.5 | 3.84 |
| 10-20 | 10YR2/2 | 4.44 | 0.09 | 0.16 | 1.93 | 4.00 | 5.41 | 4.34 | 13.36 | 6.37 |
| 20-30 | 10YR2/2 5YR6/6 2.5Y8/3 | 15.33 | 1.39 | 1.40 | 4.19 | 3.22 | 3.56 | 2.84 | 12.37 | 0.89 |
| 30-36 | 2.5Y8/3 10YR2/2 | 15.94 | 0.71 | 0.63 | 1.45 | 1.30 | 1.55 | 1.33 | 6.63 | 0.43 |
| **Core 14** | | | | | | | | | | |
| 0-10 | 10YR2/2 10YR4/6 10YR8/6 | 0.00 | 0.00 | 0.02 | 0.22 | 0.65 | 1.47 | 1.53 | 6.28 | 2.87 |
| 10-20 | 10YR3/1 10YR5/1 10YR8/6 | 0.91 | 1.18 | 0.96 | 3.90 | 6.16 | 6.35 | 5.90 | 19.57 | 2.88 |
| 20-30 | 10YR6/1  10YR7/2 | 0.33 | 0.56 | 0.54 | 2.72 | 5.52 | 7.87 | 8.05 | 24.99 | 1.98 |
| 30-40 | 10YR6/1 10YR8/1 | 0.83 | 0.38 | 0.65 | 2.58 | 4.15 | 6.91 | 5.82 | 25.58 | 1.96 |
| 40-50 | 10YR6/1 10YR8/1 | 0.84 | 0.52 | 0.67 | 2.72 | 4.12 | 6.82 | 6.14 | 21.49 | 2.93 |
| 50-60 | 10YR6/1 10YR8/1 | 0.55 | 0.49 | 0.54 | 2.08 | 3.61 | 6.25 | 5.12 | 21.41 | 1.38 |
| 60-70 | 10YR5/1 10YR5/6 | 0.67 | 0.55 | 0.85 | 3.36 | 4.29 | 5.63 | 4.60 | 14.63 | 5.99 |
| 70-80 | 10YR6/1 10YR7/6 | 1.40 | 0.80 | 1.31 | 4.76 | 4.90 | 6.52 | 5.74 | 19.10 | 3.94 |
| 80-90 | 10YR6/1 10YR8/1 | 0.76 | 0.56 | 0.70 | 3.10 | 4.79 | 6.31 | 5.34 | 18.51 | 1.94 |
| 90-100 | 10YR6/1 10YR2/1 | 0.10 | 0.26 | 0.73 | 3.07 | 5.70 | 7.23 | 6.58 | 21.72 | 3.12 |
| 100-110 | 10YR6/1 | 0.44 | 0.65 | 0.97 | 3.89 | 6.31 | 8.49 | 7.13 | 21.69 | 3.01 |
| 110-120 | 10YR6/1 5YR6/6 | 0.27 | 0.46 | 0.79 | 3.46 | 5.70 | 8.53 | 6.47 | 19.64 | 7.41 |
| 120-130 | 10YR4/1 10YR5/1 10YR6/1 | 0.21 | 0.27 | 0.52 | 2.42 | 3.41 | 6.17 | 6.01 | 21.75 | 0.89 |
| 130-140 | 10YR4/1 10YR5/1 10YR6/1 | 0.17 | 0.21 | 0.37 | 2.20 | 3.81 | 8.01 | 6.62 | 19.66 | 6.83 |
| 140-150 | 10YR4/1 10YR5/1 10YR6/1 | 0.74 | 0.34 | 0.45 | 1.67 | 2.90 | 5.85 | 6.66 | 27.88 | 2.07 |
| 150-160 | 10YR4/1 10YR5/1 10YR6/1 | 0.55 | 0.22 | 0.54 | 2.00 | 2.90 | 5.89 | 5.96 | 25.16 | 1.72 |
| 160-170 | 10YR6/1 10YR4/1 10YR6/3 | 0.66 | 0.38 | 0.47 | 2.16 | 3.77 | 7.63 | 7.34 | 33.57 | 1.37 |
| 170-180 | 10YR6/1 10YR5/6 10YR4/1 10YR8/1 | 2.29 | 0.48 | 0.58 | 2.02 | 3.54 | 7.27 | 7.97 | 29.56 | 3.74 |
| 180-190 | 10YR4/1 10YR6/1 10YR5/6 10YR8/1 | 0.22 | 0.27 | 0.47 | 2.06 | 4.14 | 8.80 | 8.09 | 31.31 | 2.98 |
| **Core 17** | | | | | | | | | | |
| 0-10 | 2.5Y2.5/1 | 1.47 | 0.16 | 0.38 | 1.38 | 2.17 | 3.01 | 2.70 | 8.99 | 4.51 |
| 10-20 | 2.5Y4/2 2.5Y4/1 | 8.93 | 1.03 | 1.20 | 3.51 | 3.88 | 4.81 | 4.09 | 15.16 | 7.99 |
| 20-30 | 2.5Y5/3 | 6.37 | 0.57 | 0.80 | 3.61 | 4.69 | 5.59 | 4.73 | 16.65 | 8.99 |
| 30-40 | 2.5Y5/2 2.5Y5/4 2.5Y4/1 | 7.48 | 2.23 | 2.35 | 5.90 | 5.55 | 6.47 | 5.46 | 18.75 | 9.59 |
| 40-50 | 2.5Y5/3 2.5Y3/1 | 11.78 | 0.72 | 1.46 | 3.68 | 3.90 | 5.01 | 4.18 | 14.61 | 7.20 |
| 50-60 | 2.5Y6/3 2.5Y4/1  2.5Y4/4 | 12.49 | 1.52 | 1.93 | 5.21 | 5.42 | 6.41 | 5.50 | 19.93 | 8.69 |
| 60-70 | 2.5Y6/3 2.5Y4/1 2.5Y4/4 | 4.98 | 1.03 | 1.33 | 0.39 | 3.36 | 4.53 | 3.97 | 19.2 | 9.13 |
| 70-80 | 2.5Y7/3 2.5Y7/6 | 11.95 | 1.34 | 0.92 | 0.39 | 0.67 | 2.47 | 3.00 | 38.58 | 3.63 |
| 80-90 | 2.5Y7/3 2.5Y6/6  2.5Y6/1 | 11.11 | 1.10 | 1.29 | 0.33 | 1.21 | 3.46 | 5.39 | 31.86 | 7.08 |
| 90-100 | 2.5Y7/3 10YR6/8 2.5Y3/1 | 3.62 | 0.02 | 0.18 | 1.32 | 2.47 | 6.54 | 7.00 | 28.32 | 12.29 |
| 100-110 | 2.5Y7/2  10YR6/8 | 6.45 | 0.83 | 0.12 | 1.14 | 2.90 | 5.63 | 8.57 | 22.45 | 8.89 |
| 110-120 | 2.5Y7/4 10YR6/8 | 9.50 | 0.18 | 0.21 | 3.34 | 3.80 | 5.61 | 5.34 | 21.42 | 7.78 |
| 120-130 | 2.5Y7/3 10YR6/8 2.5Y3/1 | 9.89 | 0.20 | 0.06 | 0.09 | 0.57 | 3.23 | 4.52 | 25.03 | 9.70 |
| 130-140 | 2.5Y7/3 | 6.49 | 0.05 | 0.05 | 0.65 | 2.04 | 5.49 | 5.75 | 30.37 | 11.13 |
| 140-150 | 2.5Y6/3 2.5Y3/1  2.5Y2.5/1 | 9.23 | 0.01 | 0.06 | 0.86 | 2.86 | 7.37 | 6.25 | 27.12 | 8.02 |
| 150-160 | 2.5Y7/4 2.5Y7/6 2.5Y7/2 | 8.21 | 0.17 | 0.17 | 1.35 | 2.91 | 6.38 | 5.28 | 31.05 | 7.74 |
| 160-170 | 2.5Y5/1 | 0.60 | 0.06 | 0.10 | 0.25 | 0.86 | 2.84 | 4.19 | 25.60 | 7.78 |
| 170-180 | 2.5Y5/1 | 2.16 | 0.00 | 0.05 | 1.36 | 1.49 | 3.74 | 4.05 | 25.03 | 2.70 |
| 180-190 | 2.5Y4/1 | 0.60 | 0.00 | 0.00 | 0.43 | 0.84 | 2.32 | 2.93 | 30.57 | 12.15 |
| **Core 19** | | | | | | | | | | |
| 0-10 | 10YR2/1 10YR3/4 10YR4/6 10YR4/2 | 0.79 | 1.08 | 1.72 | 4.84 | 3.15 | 2.13 | 1.16 | 2.06 | 0.27 |
| 10-20 | 10YR5/1 10YR5/2 10YR4/2 | 7.69 | 4.51 | 3.11 | 7.55 | 3.89 | 2.90 | 1.66 | 3.08 | 0.36 |
| 20-30 | 10YR5/1 10YR6/1 10YR7/1 | 0.40 | 0.51 | 1.12 | 4.51 | 5.23 | 6.09 | 4.17 | 11.19 | 5.13 |
| 30-40 | 10YR5/1 10YR6/1 10YR7/1 | 1.49 | 1.22 | 2.17 | 6.77 | 5.76 | 5.78 | 3.81 | 9.95 | 4.69 |
| 40-50 | 10YR5/1 10YR6/1 10YR7/1 | 1.39 | 1.21 | 1.63 | 5.41 | 6.15 | 6.92 | 5.65 | 15.36 | 6.76 |
| 57-60 | 10YR5/1 10YR6/1 10YR7/1 | 0.03 | 0.15 | 0.19 | 1.15 | 1.55 | 1.86 | 1.44 | 5.91 | 0.23 |
| 60-70 | 10YR5/1 10YR6/1 10YR7/1 | 0.42 | 0.73 | 1.33 | 5.94 | 5.89 | 6.38 | 4.97 | 17.79 | 0.85 |
| 70-80 | 10YR5/1 10YR6/1 10YR7/1 10YR7/2 | 3.84 | 2.19 | 3.05 | 7.72 | 5.54 | 5.13 | 3.33 | 8.33 | 3.56 |
| 80-90 | 10YR6/2  10YR7/2 10YR6/1 | 0.66 | 0.87 | 1.53 | 5.75 | 5.89 | 7.01 | 5.12 | 15.30 | 9.18 |
| 90-100 | 5Y7/2 5Y6/1 10YR6/6 10YR6/2 10YR7/2 | 9.35 | 4.14 | 3.88 | 6.62 | 4.06 | 3.49 | 2.36 | 9.94 | 0.52 |
| 100-110 | 5Y7/2 10YR6/8 5Y2.5/1 | 0.85 | 1.11 | 1.12 | 6.75 | 5.56 | 5.66 | 3.87 | 13.84 | 1.20 |
| 110-120 | 5Y8/2 5Y7/2 10YR6/8 5Y2.5/1 | 1.54 | 0.93 | 1.34 | 6.21 | 5.52 | 5.69 | 4.76 | 11.9 | 0.41 |
| **Core 20** | | | | | | | | | | |
| 0-10 | 2.5Y2.5/1 2.5Y3/1 | 0.12 | 0.45 | 0.85 | 4.54 | 4.28 | 4.22 | 2.59 | 7.71 | 0.36 |
| 10-20 | 2.5Y2.5/1 2.5Y3/1 | 0.67 | 1.29 | 1.20 | 3.99 | 2.77 | 2.57 | 1.40 | 3.46 | 0.21 |
| 20-30 | 2.5Y4/1 2.5Y5/1 2.5Y2.5/1 | 1.39 | 0.89 | 1.34 | 4.70 | 4.79 | 5.48 | 3.94 | 12.06 | 5.18 |
| 30-40 | 2.5Y6/1 2.5Y7/2 2.5Y7/1 | 0.90 | 0.76 | 1.60 | 5.37 | 5.40 | 5.70 | 4.76 | 13.18 | 7.36 |
| 40-50 | 2.5Y6/1 2.5Y7/2 2.5Y7/1 | 1.06 | 1.05 | 1.71 | 5.88 | 5.47 | 6.20 | 4.50 | 12.99 | 6.09 |
| 50-60 | 2.5Y6/1 2.5Y7/2  2.5Y7/1 2.5Y8/1 | 2.12 | 2.34 | 4.17 | 7.82 | 5.76 | 6.05 | 4.07 | 15.51 | 0.76 |
| 60-70 | 2.5Y6/1 2.5Y5/1 2.5Y7/1 | 8.90 | 3.39 | 2.66 | 5.51 | 3.57 | 3.68 | 2.86 | 12.6 | 0.70 |
| 70-80 | 2.5Y6/2 2.5Y5/1 2.5Y7/1 | 14.26 | 2.86 | 2.47 | 4.87 | 3.39 | 3.51 | 2.94 | 11.05 | 0.38 |
| 80-90 | 2.5Y6/2 2.5Y5/1 2.5Y8/3 | 0.39 | 0.18 | 0.54 | 3.66 | 5.43 | 6.89 | 5.48 | 17.33 | 10.53 |
| 90-100 | 2.5Y6/2 2.5Y6/3 2.5Y8/3 2.5Y2.5/1 | 0.54 | 0.78 | 1.16 | 4.84 | 5.23 | 6.75 | 5.43 | 16.96 | 12.18 |
| 100-110 | 5Y6/2  5Y6/3 5Y8/1 2.5Y6/2 2.5Y2.5/1 | 9.86 | 3.24 | 2.77 | 6.60 | 4.75 | 5.08 | 3.86 | 15.68 | 1.39 |
| 110-120 | 5Y6/2  5Y6/3 5Y7/3 5Y8/1 5Y2.5/1 | 6.27 | 3.57 | 2.93 | 6.17 | 3.84 | 3.86 | 2.96 | 11.41 | 0.38 |
| 120-130 | 5Y7/3 5Y6/2 5Y6/3 5Y8/1 5Y6/1 | 7.78 | 2.55 | 1.78 | 4.66 | 3.14 | 3.27 | 2.26 | 7.59 | 0.22 |

**3. Radiocarbon age determinations**

*3.1 Radiocarbon sample collection*

Over a two-year period, 2009 and 2010, AMS radiocarbon samples (i.e., carbonized plant remains) were collected from excavation units and wet and dry cores in the Corriental, Perdido, and Temple reservoirs and the Inscriptions sinkhole to evaluate their chronostratigraphy and hydrology and their relationship to the occupation of Tikal. A hand-operated JMC percussion soil sampler was used to extract solid sediment cores from the Corriental, Perdido, and Temple reservoirs and the Inscriptions sinkhole. Two-cm diameter and 1 m-long stainless-steel core-tubes were pounded into the ground by hand using a 5.0 kg slide-hammer to a depth of 3.0 m or to refusal. Samples were collected directly into clear, PETG co-polyester liners with red (top) and black (bottom) color-coded vinyl caps. Excavations were hand dug and sedimentary units were numbered based on their stratigraphic sequence and soils were characterized using Munsell soil color, sedimentary structures, and particle size.

*3.2 Laboratory analysis of radiocarbon samples*

Seven radiocarbon samples, four from the Inscriptions sinkhole and three from the Temple reservoir, were submitted to and processed at the National Ocean Sciences Accelerator Mass Spectrometry (NOSAMS) facility. Nine radiocarbon samples, six from the Corriental reservoir, two from the Perdido reservoir, and one from the Temple reservoir, were submitted to the Beta Analytic Testing Laboratory.

Samples were placed in a 100 ml glass beaker with pure water. A small magnetic stirrer was used to gently agitate the sample to remove sediment matrix adhering to the sample. Aliquots of exemplary carbon were hand selected. A dental probe, tweezers, and a scalpel were used to physically separate and remove uncarbonized organic materials such as rootlets and inorganic materials such as carbonate sediment under an optical binocular microscope.

Radiocarbon samples were subjected to a standard acid-base-acid (ABA) pretreatment. They were initially washed with in 1 N HCl at 70 °C for 30 minutes to dissolve all carbonates. The remaining sample was washed in 1 N NaOH at 70 °C for 30 min to remove organic acids. NaOH washing was repeated until the solution was clear. A final rinse in 1 N HCl for 30 minutes neutralized the NaOH. The resulting radiocarbon samples were washed in pure water until a neutral pH was obtained.

Age determinations were made using an accelerator mass spectrometer (AMS) with two Source of Negative Ions by Cesium Sputtering ion sources (SNICS). An accurate total fractionation correction was obtained using a sequential injection of ^13^C and ^12^C for the ^13^C/^12^C ratio within the instrument. The radiocarbon age calculations were based on both the ^14^C/^12^C and ^14^C/^13^C ratios using the sequential injection of ^14^C, ^13^C, and ^12^C to provide three different measurements to ensure a stable isotope pathway during the analysis. The ^13^C/^12^C ratio was measured to correct for the total fractionation and additionally analyzed in a Thermo-Finnegan Delta Plus Isotope Ratio Mass Spectrometer (IRMS). A modern standard of oxalic acid was used to calculate a fraction of modern value for the control. Multiple background measurements are made before and after of each analysis to ensure the absence of inter-sample contamination. Four to five modern standards of known-age were run to evaluate the accuracy the unknown sample ages.

**table S4. AMS radiocarbon ages from Maya reservoir and sinkhole sediments at Tikal, Guatemala.**

| **Lab**  **Number** | **Sample**  **Composition** | | | | **Provenience** | | | **Depth**  **(cm)** | | | **Measured ^14^C Age yr. B.P. (1 σ)** | | **Calibrated ^14^C Age yr. B.P. (2 σ)** | | | **Probability (95%)** |
| --- | --- | --- | --- | --- | --- | --- | --- | --- | --- | --- | --- | --- | --- | --- | --- | --- |
| **Corriental Reservoir** | | | | | | | | | | | | | | | | |
| Beta  258720^a^ | Charcoal | | | | Op 1C | | | 65-80 | | | 990 + 40 | | 965-795 | | | 965-795 (95.4%) |
| Beta  280839^b^ | SOM | | | | Op 1L,  Core 8 | | | 140-180 | | | 2,010 + 40 | | 2105-1875 | | | 2105-2086 (2.0%)  2063-1875 (93.4%) |
| Beta  266124^a^ | SOM | | | | Op 1C | | | 162-194 | | | 2,110 + 40 | | 2300-1953 | | | 2300-2251 (6.7%)  2159-1987 (88.5%)  1957-1953 (0.3%) |
| Beta  280837^b^ | SOM | | | | Op 1L,  Core 8 | | | 180-230 | | | 2,120 + 40 | | 2301-2161 | | | 2301-2246 (10.6%)  2178-2170 (0.8%)  2161-1992 (84.1%) |
| Beta  258721^a^ | SOM | | | | Op 1C | | | 265-290 | | | 2,340 + 40 | | 2671-2185 | | | 2671-2670 (0.1%)  2652-2645 (0.4%)  2490-2306 (91.7%)  2232-2204 (2.5%)  2194-2185 (0.6%) |
| Beta  270566^b^ | SOM | | | | Op 1L,  Core 8 | | | 310-312 | | | 8,960 + 60 | | 10,233-9110 | | | 10,233-9110 (95.4%) |
| **Inscriptions Sinkhole** | | | | | | | | | | | | | | | | |
| NOSAMS  88678^b^ | | SOM | | Op 1L, Core 20-1 | | | 50-60 | | | 4,170 + 35 | | 4834-4581 | | | 4834-4780 (20.3%)  4770-4581 (75.1%) | |
| NOSAMS  88678^b^ | | SOM | | Op 1L, Core 20-2 | | | 80-90 | | | 3,840 + 40 | | 4410-4103 | | | 4410-4148 (94.7%)  4108-4103 (0.7%) | |
| NOSAMS  88680^b^ | | SOM | | Op 1L, Core 20-2 | | | 90-100 | | | 3,000 + 65 | | 3360-2997 | | | 3360-2997 (95.4%) | |
| NOSAMS  88681^b^ | | SOM | | Op 1L, Core 20-1 | | | 120-130 | | | 11,600 + 100 | | 13,706-13,221 | | | 13,706-13,675 (1.2%)  13,625-13,221 (94.2) | |
| **Perdido Reservoir** | | | | | | | | | | | | | | | | |
| Beta  289287^b^ | | SOM | Op 8, Core N2E0 | | | 50-60 | | | 2,220 + 60 | | | 2350-2105 | | | 2350-2105 (93.7%)  2084-2064 (1.7%) | |
| Beta  280828^a^ | | SOM | Op 8A | | | 110 | | | 1,540 + 40 | | | 1529-1350 | | | 1529-1350 (95.4%) | |
| **Temple Reservoir** | | | | | | | | | | | | | | | | |
| Beta  281746^a^ | | Charcoal | Op 7C | | | 110 | | | 1,200 + 40 | | | 1261-1001 | | | 1261-1200 (12.9%)  1190-1050 (77.9%)  1029-1001 (4.6%) | |
| NOSAMS  85584^a^ | | SOM | Op 7C | | | 130-140 | | | 1,230 + 25 | | | 1260-1069 | | 1260-1201 (32.9%)  1190-1069 (62.5%) | | |
| NOSAMS  85585^a^ | | SOM | Op 7C | | | 140-162 | | | 1,830 + 25 | | | 1858-1705 | | 1858-1853 (0.7%)  1826-1705 (94.7%) | | |
| NOSAMS  85583^a^ | | SOM | Op 7C | | | 162-194 | | | 1,250 + 35 | | | 1277-1075 | | 1277-1170 (69.6%)  1163-1075 (25.8%) | | |

1. Sample collected from an excavation profile.
2. Dry core with some compression.
3. Wet core with compression.

**4. Mineralogy**

Multiple proxies were used to examine the mineralogy of Tikal’s reservoir and sinkhole sediments and the coarse crystalline tuff from the Bajo de Azúcar including high magnification optical microscopy, scanning electron microscopy, and X-ray diffraction analysis.

*4.1 Optical microscopy*

Sediment samples were initially wet sieved between 74 and 250 μm and subjected to LST (lithium metatungstate and diiodomethane) heavy liquid density separation to obtain distinctive volcanogenic mineral crystals. Handpicked aliquots from reservoir and sinkhole sediments and coarse crystalline tuff from the Bajo de Azúcar were examined and isolated grains were photographed with a high magnification Leica MZ12 stereomicroscope (640x).


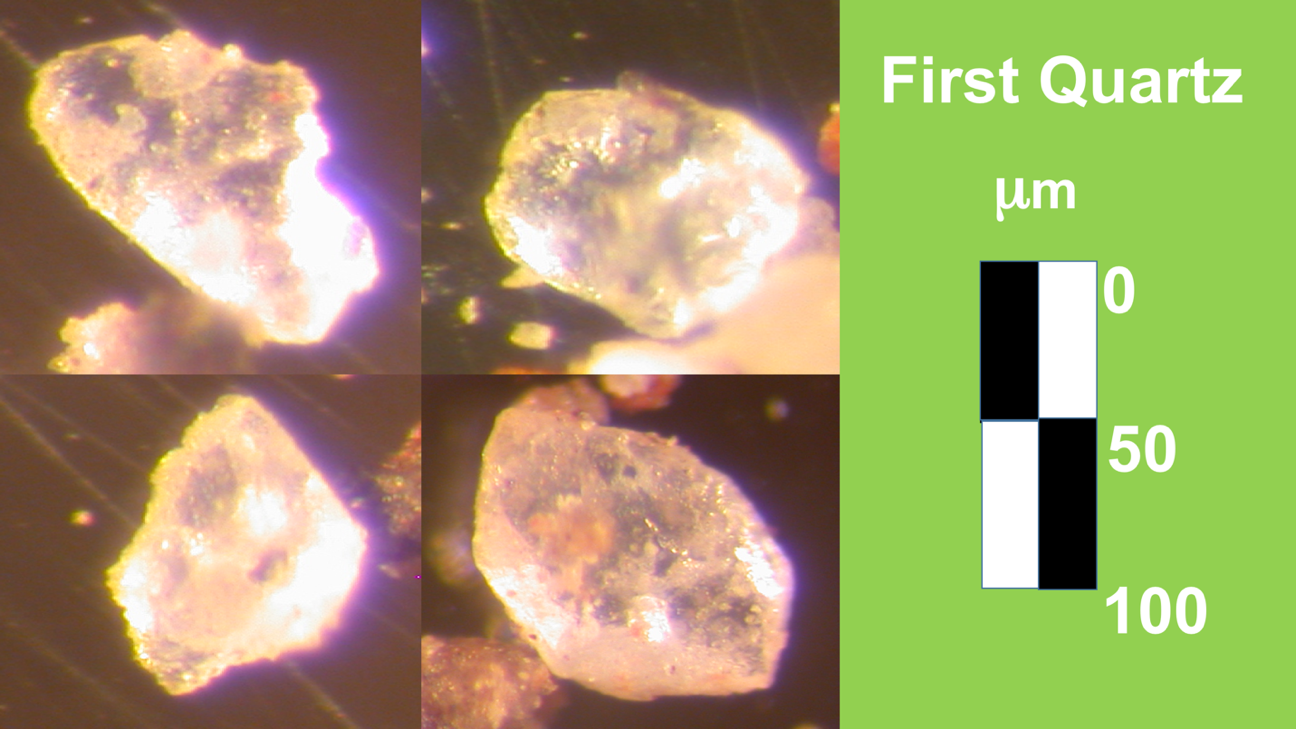


**fig. S4. Photomicrographs of volcanogenic bipyramidal euhedral “first quartz” crystals from the Corriental reservoir sediments.** Kenneth Barnett Tankersley used Microsoft PowerPoint for Mac Version 16.41 ([www.microsoft.com](http://www.microsoft.com)) to create this figure.


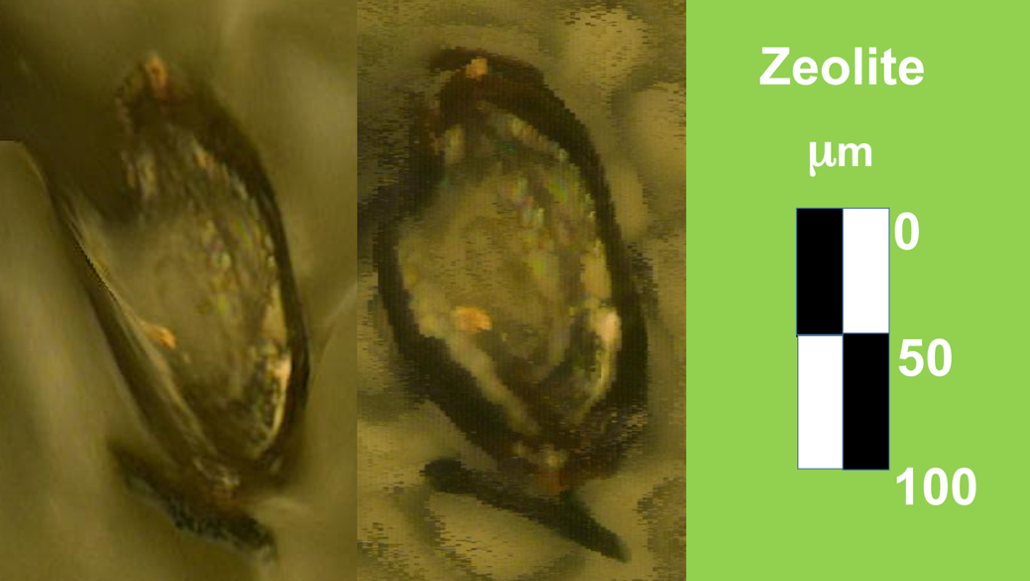


**fig. S5. Photomicrographs of volcanogenic zircon crystals from the Corriental reservoir.** Kenneth Barnett Tankersley used Microsoft PowerPoint for Mac Version 16.41 ([www.microsoft.com](http://www.microsoft.com)) to create this figure.


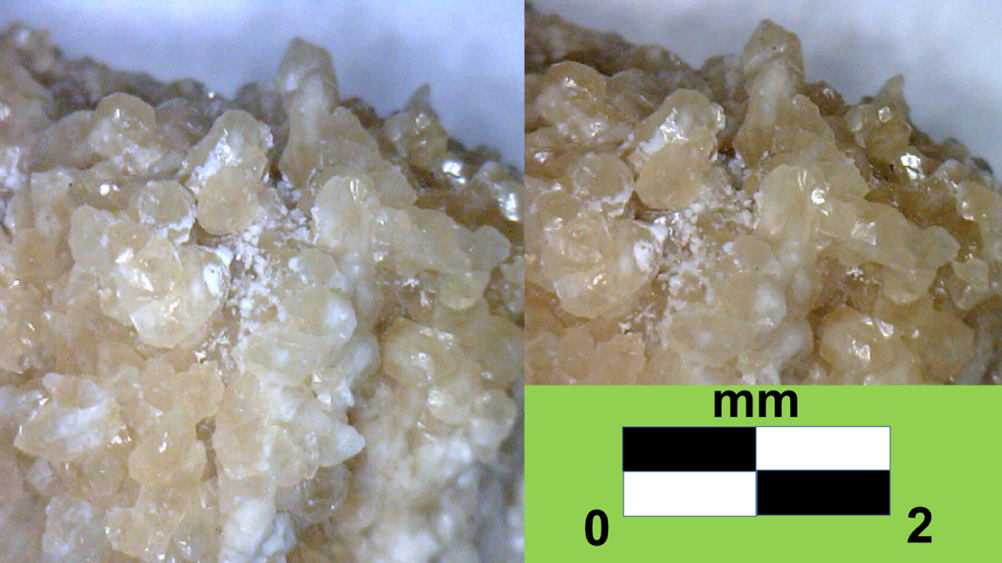


**fig. S6. Photomicrographs of coarse crystalline tuff (euhedral quartz = amber, zeolite = white—clinoptilolite and mordenite) from the Bajo de Azúcar located ~30 km northeast of Tikal.** Kenneth Barnett Tankersley used Microsoft PowerPoint for Mac Version 16.41 ([www.microsoft.com](http://www.microsoft.com)) to create this figure.


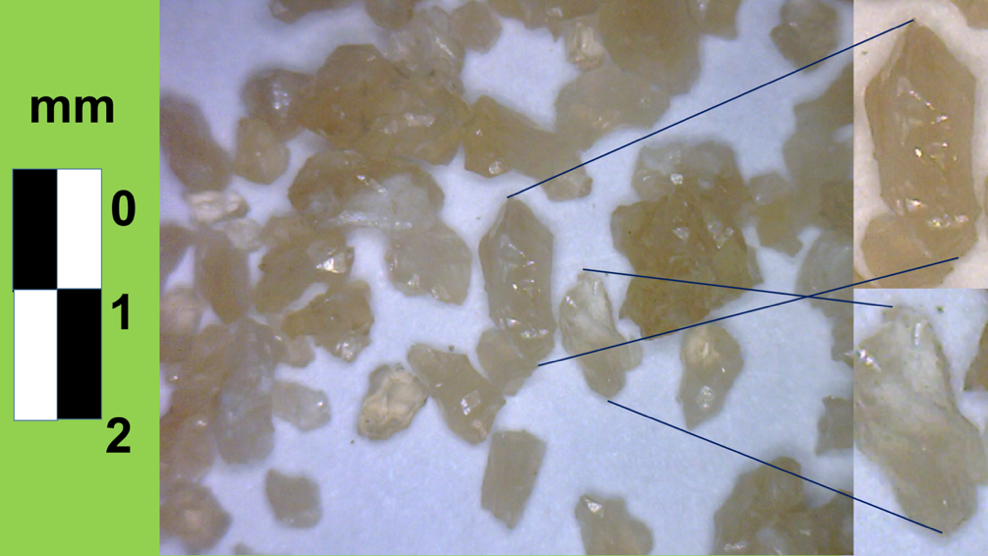


**fig. S7. Photomicrographs of anthropogenic macrocrystalline euhedral quartz crystals from the Corriental reservoir sediments.** Kenneth Barnett Tankersley used Microsoft PowerPoint for Mac Version 16.41 ([www.microsoft.com](http://www.microsoft.com)) to create this figure.

*4.2 Scanning electron microscopy (SEM)*

Individual volcanogenic crystalline minerals (e.g., microcrystalline quartz and zircons) isolated by LST were examined using a SCIOS dual-beam scanning electron microscope (SEM) and focused ion beam for high resolution imaging of the mineral samples. The SEM included an in-lens (T1, T2), CBS/ABS (backscatter), ETD (secondary), STEM with HAADF imaging detectors, FIB milling with Gallium LMIS, platinum deposition, EDX elemental analysis, EBSD for grain orientation mapping, and E-beam lithography.


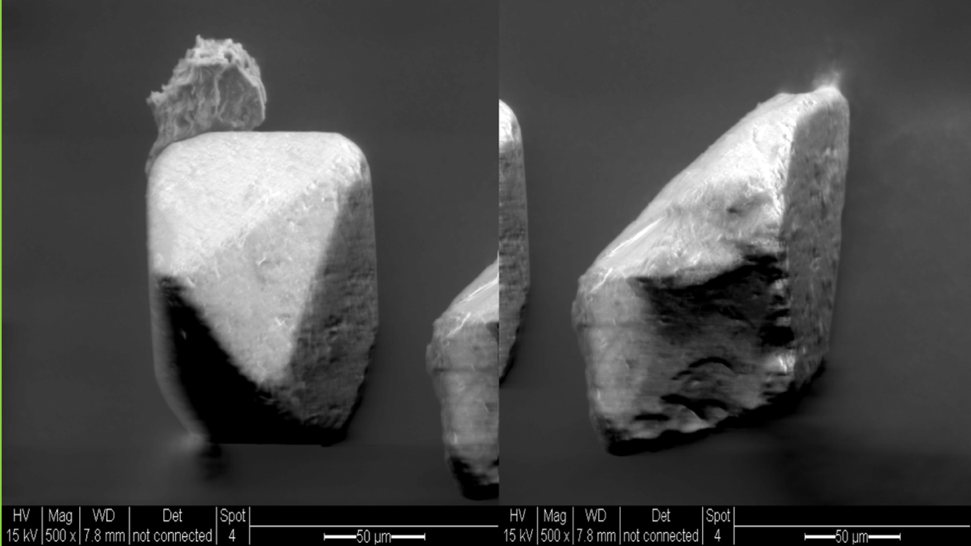


**fig. S8 Scanning electron micrographs of volcanogenic bipyramidal euhedral quartz crystals from the Corriental reservoir sediments.** Kenneth Barnett Tankersley used Microsoft PowerPoint for Mac Version 16.41 ([www.microsoft.com](http://www.microsoft.com)) to create this figure.


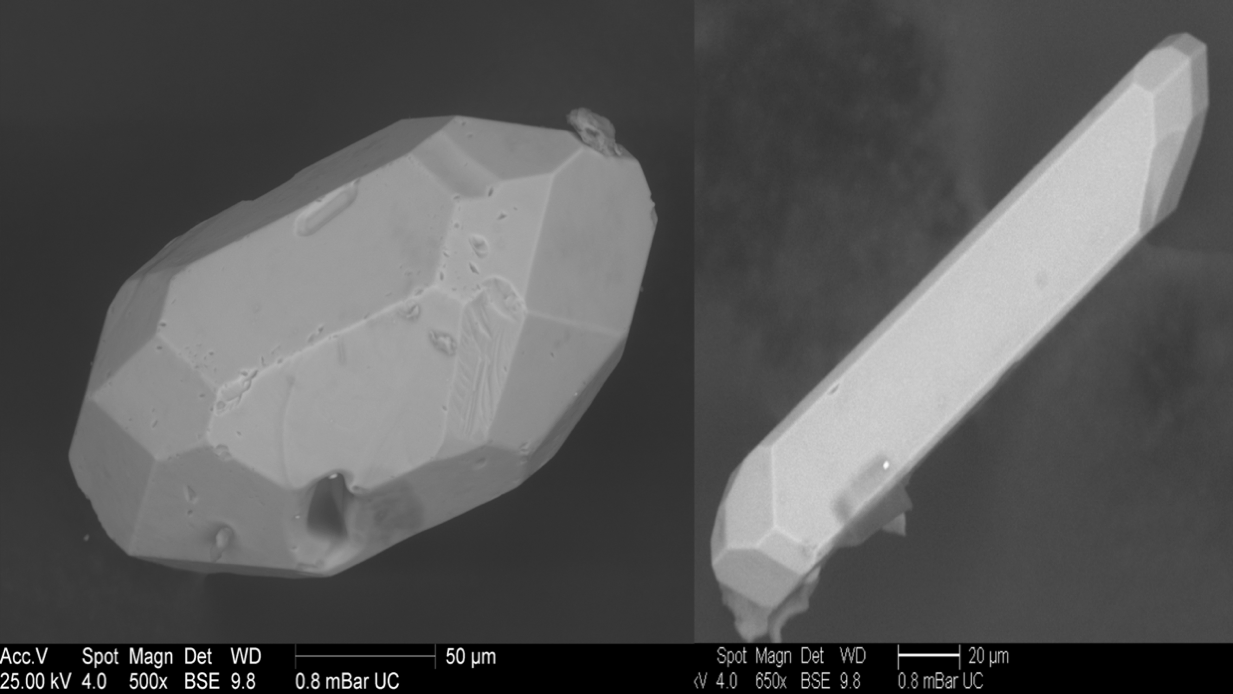


**fig. S9. Scanning electron micrographs of volcanogenic zircon crystals from the Corriental reservoir sediments.** Kenneth Barnett Tankersley used Microsoft PowerPoint for Mac Version 16.41 ([www.microsoft.com](http://www.microsoft.com)) to create this figure.

*4.3 Powder X-ray diffraction analysis (XRD)*

Sediments samples (20 g), which were collected at 10-cm intervals, were initially sieved through a 2 mm mesh. A slurry was then made from each aliquot using deionized water in a 100 ml beaker. A high-speed stirrer combined with gravity settling was used to disperse the clay fraction (<2 mm). The sample was transferred to a glass slide using a 5 ml pipette and air-dried. A duplicate sample was prepared and equilibrated for 12 hours in a bell jar with ethylene glycol vapor. These samples were prepared to determine the presence of expandable clay minerals. Both the air-dried and glycolated samples were subjected to X-ray diffraction analysis (XRD).

Sediment samples were analyzed on a Siemens D-500 X-ray diffractometer using a Cu-Kα radiation source. Each slide was scanned from 2^o^ to 32^o^ 2θ at 0.5 increments and then broadened to 60^o^ 2θ and intensity threshold was set at 1.6. Peak position and peak intensity were used to identify the mineralogical composition of the samples (S12). Relative percent mineral compositions were calculated from the total counts per second (cps). The peak sum cps for each mineral was divided by the total cps.

**table S5. Relative percent mineral composition of Tikal reservoirs and sinkhole (control) sediments based on powder X-ray diffraction (XRD) analysis.**

| **Depth (cm)** | **Calcite (%)** | **Smectite (%)** | **Quartz (%)** | **Zeolite (%)** |
| --- | --- | --- | --- | --- |
| **Corriental Reservoir** | | | | |
| 0-10 | 31 | 18 | 37 | 13 |
| 10-20 | 66 | 10 | 10 | 7 |
| 20-30 | 68 | 6 | 11 | 4 |
| 30-40 | 76 | 7 | 12 | 5 |
| 40-50 | 80 | 5 | 7 | 4 |
| 50-60 | 70 | 9 | 13 | 6 |
| 60-70 | 86 | 5 | 6 | 3 |
| 70-80 | 75 | 7 | 7 | 5 |
| 80-90 | 47 | 22 | 36 | 16 |
| 90-100 | 89 | 1 | 4 | 1 |
| 100-110 | 81 | 9 | 5 | 6 |
| 120-130 | 68 | 7 | 3 | 6 |
| 130-140 | 52 | 5 | 4 | 4 |
| 140-150 | 74 | 5 | 3 | 4 |
| 150-160 | 71 | 8 | 7 | 6 |
| 160-170 | 20 | 42 | 3 | 32 |
| 170-180 | 18 | 23 | 6 | 17 |
| 190-200 | 79 | 7 | 10 | 5 |
| 200-210 | 68 | 3 | 4 | 2 |
| 220-230 | 80 | 6 | 5 | 4 |
| 230-240 | 70 | 10 | 11 | 8 |
| 240-250 | 84 | 11 | 12 | 9 |
| 250-260 | 84 | 3 | 4 | 1 |
| 260-270 | 79 | 6 | 2 | 4 |
| 270-280 | 92 | 4 | 5 | 3 |
| 280-290 | 76 | 6 | 12 | 4 |
| 290-300 | 64 | 11 | 12 | 8 |
| 300-310 | 91 | 9 | 4 | 7 |
| **Inscriptions Sinkhole** | | | | |
| 0-10 | 0 | 76 | 24 | 0 |
| 10-20 | 0 | 84 | 16 | 0 |
| 20-30 | 61 | 26 | 13 | 0 |
| 30-40 | 60 | 29 | 11 | 0 |
| 40-50 | 64 | 27 | 9 | 0 |
| 60-70 | 70 | 23 | 7 | 0 |
| 70-80 | 66 | 25 | 9 | 0 |
| 100-110 | 80 | 12 | 8 | 0 |
| 110-120 | 59 | 23 | 18 | 0 |
| **Perdido Reservoir** | | | | |
| 0-10 | 93 | 4 | 3 | 0 |
| 10-20 | 97 | 1 | 3 | 0 |
| 20-30 | 53 | 39 | 4 | 0 |
| 30-40 | 94 | 1 | 5 | 0 |
| 40-50 | 80 | 16 | 4 | 0 |
| 50-60 | 94 | 3 | 4 | 0 |
| 60-70 | 95 | 1 | 4 | 0 |
| 70-80 | 92 | 5 | 3 | 0 |
| 80-90 | 97 | 1 | 2 | 0 |
| 90-100 | 74 | 21 | 5 | 0 |
| 100-110 | 97 | 1 | 2 | 0 |
| 110-120 | 69 | 25 | 6 | 0 |
| 120-130 | 48 | 38 | 14 | 0 |
| 130-140 | 98 | 1 | 1 | 0 |
| 140-150 | 48 | 38 | 14 | 0 |
| 150-160 | 98 | 1 | 1 | 0 |
| 160-170 | 26 | 54 | 20 | 0 |
| 170-180 | 38 | 54 | 8 | 0 |
| 180-190 | 60 | 21 | 19 | 0 |
| 190-200 | 67 | 23 | 10 | 0 |
| 200-210 | 51 | 39 | 10 | 0 |
| 210-220 | 71 | 23 | 6 | 0 |
| 220-230 | 60 | 30 | 10 | 0 |
| 230-240 | 42 | 52 | 6 | 0 |
| 240-250 | 43 | 46 | 11 | 0 |
| 250-260 | 34 | 54 | 12 | 0 |
| 260-270 | 7 | 84 | 9 | 0 |
| 270-280 | 25 | 59 | 16 | 0 |
| 280-290 | 11 | 78 | 11 | 0 |
| 290-300 | 37 | 48 | 15 | 0 |
| 300-310 | 61 | 25 | 14 | 0 |
| 310-320 | 36 | 41 | 23 | 0 |
| 320-330 | 38 | 52 | 10 | 0 |
| 330-340 | 35 | 34 | 31 | 0 |
| 340-350 | 26 | 65 | 9 | 0 |
| 350-360 | 33 | 62 | 5 | 0 |
| 360-370 | 21 | 67 | 12 | 0 |

| **Temple Reservoir, Main Tank** | | | | |
| --- | --- | --- | --- | --- |
| 0-10 | 83 | 13 | 4 | 0 |
| 10-20 | 81 | 14 | 5 | 0 |
| 20-30 | 89 | 11 | 0 | 0 |
| 30-40 | 85 | 9 | 3 | 0 |
| 40-50 | 85 | 8 | 7 | 0 |
| 50-60 | 89 | 9 | 2 | 0 |

**table S6. X-ray diffraction wavelength peaks in angstroms (Å) and their associated crystalline minerals per depth from the Corriental Reservoir.**

| **Mineral** | **Depth**  **(cm)** | **XRD Peak**  **(Å)** | **Length (mm)** | **Height**  **(mm)** | **Area**  **(mm^2^)** |
| --- | --- | --- | --- | --- | --- |
| Smectite, Zeolite | 70-80 | 15.63 | 5.96 | 2.40 | 14.30 |
| Smectite, Zeolite | 70-80 | 5.98 | 3.22 | 1.66 | 5.35 |
| Smectite, Zeolite, Quartz | 70-80 | 4.50 | 5.59 | 2.97 | 16.60 |
| Calcite | 70-80 | 3.88 | 1.41 | 3.64 | 5.13 |
| Quartz | 70-80 | 3.37 | 1.45 | 4.74 | 6.87 |
| Calcite | 70-80 | 3.06 | 2.62 | 54.10 | 141.74 |
| Calcite | 70-80 | 2.50 | 2.34 | 7.35 | 17.20 |
| Calcite | 70-80 | 2.29 | 2.28 | 23.20 | 52.90 |
| Calcite | 70-80 | 1.92 | 2.31 | 9.47 | 21.88 |
| Calcite | 70-80 | 1.88 | 1.68 | 9.49 | 15.94 |
| Calcite | 70-80 | 1.61 | 1.42 | 2.96 | 4.20 |
| Smectite, Zeolite | 60-70 | 15.63 | 4.25 | 1.61 | 6.84 |
| Smectite, Zeolite | 60-70 | 4.50 | 2.54 | 1.89 | 4.80 |
| Calcite | 60-70 | 3.88 | 1.23 | 1.97 | 2.42 |
| Quartz | 60-70 | 3.37 | 1.20 | 2.41 | 2.89 |
| Calcite | 60-70 | 3.06 | 2.15 | 37.58 | 80.80 |
| Calcite | 60-70 | 2.50 | 1.62 | 2.92 | 4.73 |
| Calcite | 60-70 | 2.29 | 1.72 | 4.47 | 7.69 |
| Calcite | 60-70 | 1.92 | 2.37 | 6.20 | 14.69 |
| Calcite | 60-70 | 1.88 | 1.40 | 4.78 | 6.69 |
| Calcite | 60-70 | 1.61 | 0.91 | 3.36 | 3.06 |
| Smectite, Zeolite | 50-60 | 15.63 | 5.85 | 2.24 | 13.10 |
| Smectite, Zeolite | 50-60 | 5.98 | 2.24 | 2.09 | 4.68 |
| Smectite, Zeolite | 50-60 | 4.50 | 4.30 | 4.95 | 21.29 |
| Calcite | 50-60 | 3.88 | 2.04 | 6.29 | 12.83 |
| Quartz | 50-60 | 3.37 | 2.14 | 6.15 | 13.16 |
| Calcite | 50-60 | 3.06 | 2.10 | 30.44 | 63.92 |
| Calcite | 50-60 | 2.50 | 1.87 | 8.15 | 15.24 |
| Calcite | 50-60 | 2.29 | 1.83 | 11.82 | 21.63 |
| Calcite | 50-60 | 1.92 | 3.04 | 12.46 | 37.88 |
| Calcite | 50-60 | 1.88 | 1.77 | 13.77 | 24.37 |
| Calcite | 50-60 | 1.61 | 2.19 | 5.01 | 10.97 |
| Smectite, Zeolite | 40-50 | 15.63 | 2.42 | 2.11 | 5.11 |
| Smectite, Zeolite | 40-50 | 5.98 | 2.76 | 1.88 | 5.19 |
| Smectite, Zeolite | 40-50 | 4.50 | 3.04 | 3.87 | 11.76 |
| Calcite | 40-50 | 3.88 | 1.71 | 7.89 | 13.49 |
| Quartz | 40-50 | 3.37 | 1.88 | 3.34 | 6.28 |
| Calcite | 40-50 | 3.06 | 2.49 | 35.14 | 87.50 |
| Calcite | 40-50 | 2.50 | 1.76 | 7.79 | 13.71 |
| Calcite | 40-50 | 2.29 | 2.13 | 11.79 | 25.11 |
| Calcite | 40-50 | 1.92 | 2.08 | 11.36 | 23.63 |
| Calcite | 40-50 | 1.88 | 1.38 | 16.76 | 23.13 |
| Calcite | 40-50 | 1.61 | 1.90 | 5.70 | 10.83 |
| Smectite, Zeolite | 30-40 | 5.98 | 2.56 | 2.96 | 7.58 |
| Smectite, Zeolite,  Quartz | 30-40 | 4.50 | 3.39 | 4.11 | 13.93 |
| Calcite | 30-40 | 3.88 | 1.60 | 3.85 | 6.16 |
| Calcite | 30-40 | 3.37 | 1.47 | 4.96 | 7.29 |
| Calcite | 30-40 | 3.06 | 2.01 | 31.86 | 64.04 |
| Calcite | 30-40 | 2.50 | 1.36 | 5.35 | 7.28 |
| Calcite | 30-40 | 2.29 | 1.69 | 10.61 | 17.93 |
| Calcite | 30-40 | 1.92 | 2.44 | 7.46 | 18.20 |
| Calcite | 30-40 | 1.88 | 1.80 | 9.69 | 17.44 |
| Calcite | 30-40 | 1.61 | 1.38 | 5.35 | 7.38 |
| Smectite, Zeolite | 20-30 | 15.63 | 5.85 | 2.91 | 17.02 |
| Smectite, Zeolite, Quartz | 20-30 | 4.50 | 2.48 | 2.48 | 6.15 |
| Calcite | 20-30 | 3.88 | 1.68 | 5.14 | 8.64 |
| Quartz | 20-30 | 3.37 | 1.54 | 12.95 | 19.94 |
| Calcite | 20-30 | 3.06 | 1.97 | 34.18 | 67.33 |
| Calcite | 20-30 | 2.50 | 1.35 | 3.58 | 4.83 |
| Calcite | 20-30 | 2.29 | 1.53 | 7.70 | 11.78 |
| Calcite | 20-30 | 1.92 | 2.22 | 20.17 | 44.78 |
| Calcite | 20-30 | 1.88 | 1.37 | 7.34 | 10.06 |
| Calcite | 20-30 | 1.61 | 1.56 | 7.21 | 11.25 |
| Smectite, Zeolite | 10-20 | 15.63 | 4.92 | 3.31 | 16.29 |
| Smectite, Zeolite, Quartz | 10-20 | 4.50 | 1.19 | 5.26 | 6.26 |
| Calcite | 10-20 | 3.88 | 1.76 | 2.85 | 5.02 |
| Quartz | 10-20 | 3.37 | 1.48 | 4.80 | 7.10 |
| Calcite | 10-20 | 3.06 | 2.08 | 18.78 | 39.06 |
| Calcite | 10-20 | 2.50 | 1.41 | 3.58 | 5.05 |
| Calcite | 10-20 | 2.29 | 1.54 | 8.33 | 12.83 |
| Calcite | 10-20 | 1.92 | 1.86 | 9.76 | 18.15 |
| Calcite | 10-20 | 1.88 | 1.29 | 4.24 | 5.47 |
| Calcite | 10-20 | 1.61 | 1.25 | 2.76 | 3.45 |
| Smectite, Zeolite | 0-10 | 15.63 | 6.32 | 3.04 | 19.21 |
| Smectite, Zeolite, Quartz | 0-10 | 4.50 | 4.43 | 6.36 | 28.17 |
| Calcite | 0-10 | 3.88 | 1.68 | 3.39 | 5.70 |
| Quartz | 0-10 | 3.37 | 1.94 | 14.73 | 28.58 |
| Calcite | 0-10 | 3.06 | 1.91 | 20.27 | 38.72 |
| Calcite | 0-10 | 2.50 | 1.35 | 2.50 | 3.38 |
| Calcite | 0-10 | 2.29 | 1.57 | 4.70 | 7.38 |
| Calcite | 0-10 | 1.92 | 1.77 | 3.06 | 5.42 |
| Calcite | 0-10 | 1.88 | 1.48 | 5.60 | 8.29 |
| Calcite | 0-10 | 1.61 | 1.29 | 1.47 | 1.90 |
| Smectite, Zeolite, Quartz | 150-160 | 4.48 | 2.31 | 5.47 | 12.64 |
| Calcite | 150-160 | 3.88 | 2.21 | 5.87 | 12.97 |
| Quartz | 150-160 | 3.37 | 4.13 | 1.19 | 4.91 |
| Calcite | 150-160 | 3.05 | 2.26 | 42.45 | 95.94 |
| Calcite | 150-160 | 2.50 | 1.62 | 7.67 | 12.43 |
| Calcite | 150-160 | 2.29 | 1.53 | 12.20 | 18.67 |
| Calcite | 150-160 | 1.92 | 12.48 | 1.44 | 17.97 |
| Calcite | 150-160 | 1.88 | 1.51 | 13.16 | 19.87 |
| Calcite | 150-160 | 1.63 | 1.65 | 1.80 | 2.97 |
| Calcite | 150-160 | 1.61 | 1.31 | 5.88 | 7.70 |
| Smectite, Zeolite | 140-150 | 5.82 | 2.59 | 1.88 | 4.87 |
| Smectite,  Zeolite,  Quartz | 140-150 | 4.48 | 1.47 | 3.26 | 4.79 |
| Calcite | 140-150 | 3.88 | 1.03 | 6.07 | 6.25 |
| Quartz | 140-150 | 3.37 | 1.30 | 3.24 | 4.21 |
| Calcite | 140-150 | 3.05 | 2.26 | 63.80 | 144.19 |
| Calcite | 140-150 | 2.50 | 1.06 | 9.78 | 10.37 |
| Calcite | 140-150 | 2.29 | 1.55 | 13.93 | 21.59 |
| Calcite | 140-150 | 1.92 | 1.26 | 13.09 | 16.49 |
| Calcite | 140-150 | 1.88 | 1.04 | 13.08 | 13.60 |
| Calcite | 140-150 | 1.61 | 1.47 | 6.40 | 9.41 |
| Smectite, Zeolite | 130-140 | 5.82 | 2.42 | 2.40 | 5.81 |
| Smectite, Zeolite,  Quartz | 130-140 | 4.48 | 1.70 | 4.99 | 8.48 |
| Calcite | 130-140 | 3.88 | 1.00 | 5.29 | 5.29 |
| Quartz | 130-140 | 3.37 | 1.42 | 2.82 | 4.00 |
| Calcite | 130-140 | 3.05 | 2.61 | 38.86 | 101.42 |
| Calcite | 130-140 | 2.50 | 1.18 | 7.77 | 9.17 |
| Calcite | 130-140 | 2.29 | 1.76 | 15.38 | 27.07 |
| Calcite | 130-140 | 1.92 | 1.34 | 13.25 | 17.76 |
| Calcite | 130-140 | 1.88 | 1.65 | 15.23 | 25.13 |
| Calcite | 130-140 | 1.61 | 1.44 | 9.31 | 13.41 |
| Quartz | 120-130 | 3.37 | 1.86 | 3.47 | 6.45 |
| Calcite | 120-130 | 3.05 | 1.65 | 53.28 | 87.91 |
| Calcite | 120-130 | 2.50 | 1.01 | 7.39 | 7.46 |
| Calcite | 120-130 | 2.29 | 2.25 | 9.45 | 21.26 |
| Calcite | 120-130 | 1.92 | 2.20 | 9.03 | 19.87 |
| Calcite | 120-130 | 1.88 | 1.52 | 10.15 | 15.43 |
| Calcite | 120-130 | 1.61 | 1.31 | 9.99 | 13.09 |
| Smectite, Zeolite, Quartz | 110-120 | 4.48 | 2.27 | 3.99 | 9.06 |
| Calcite | 110-120 | 3.88 | 1.01 | 5.89 | 5.95 |
| Quartz | 110-120 | 3.37 | 1.17 | 5.14 | 6.01 |
| Calcite | 110-120 | 3.05 | 2.11 | 42.36 | 89.38 |
| Calcite | 110-120 | 2.50 | 1.04 | 10.02 | 10.42 |
| Calcite | 110-120 | 2.29 | 1.27 | 15.51 | 19.70 |
| Calcite | 110-120 | 1.92 | 2.31 | 15.01 | 34.67 |
| Calcite | 110-120 | 1.88 | 1.76 | 21.10 | 37.14 |
| Calcite | 110-120 | 1.61 | 1.47 | 7.97 | 11.72 |
| Calcite | 100-110 | 3.88 | 1.76 | 5.05 | 8.89 |
| Quartz | 100-110 | 3.37 | 1.57 | 1.25 | 1.96 |
| Calcite | 100-110 | 3.05 | 2.11 | 42.85 | 90.41 |
| Calcite | 100-110 | 2.50 | 1.71 | 12.15 | 20.78 |
| Calcite | 100-110 | 2.29 | 2.05 | 12.77 | 26.18 |
| Calcite | 100-110 | 1.92 | 2.16 | 9.62 | 20.78 |
| Calcite | 100-110 | 1.88 | 1.59 | 7.91 | 12.58 |
| Calcite | 100-110 | 1.61 | 1.74 | 7.32 | 12.74 |
| Smectite, Zeolite, Quartz | 90-100 | 4.48 | 2.60 | 1.76 | 4.58 |
| Calcite | 90-100 | 3.88 | 0.97 | 8.11 | 7.87 |
| Quartz | 90-100 | 3.37 | 1.98 | 2.85 | 5.64 |
| Calcite | 90-100 | 3.05 | 2.08 | 41.35 | 86.01 |
| Calcite | 90-100 | 2.50 | 1.22 | 17.63 | 21.51 |
| Calcite | 90-100 | 2.29 | 1.53 | 22.80 | 34.88 |
| Calcite | 90-100 | 1.92 | 1.76 | 12.12 | 21.33 |
| Calcite | 90-100 | 1.88 | 1.47 | 26.10 | 38.37 |
| Calcite | 90-100 | 1.61 | 1.38 | 9.76 | 13.47 |
| Smectite,  Zeolite | 80-90 | 5.82 | 3.86 | 1.27 | 4.90 |
| Smectite, Zeolite, Quartz | 80-90 | 4.48 | 2.60 | 47.38 | 123.19 |
| Calcite | 80-90 | 3.88 | 0.95 | 9.06 | 8.61 |
| Quartz | 80-90 | 3.37 | 1.11 | 7.42 | 8.24 |
| Calcite | 80-90 | 3.05 | 1.69 | 61.05 | 103.17 |
| Calcite | 80-90 | 2.86 | 1.12 | 1.99 | 2.23 |
| Calcite | 80-90 | 2.5 | 0.78 | 7.28 | 5.68 |
| Calcite | 80-90 | 2.29 | 1.27 | 13.58 | 17.25 |
| Calcite | 80-90 | 1.92 | 1.49 | 14.19 | 21.14 |
| Calcite | 80-90 | 1.88 | 1.24 | 13.26 | 16.44 |
| Calcite | 80-90 | 1.61 | 1.27 | 5.57 | 7.07 |
| Smectite,  Zeolite | 230-240 | 6.00 | 2.46 | 1.94 | 4.77 |
| Smectite, Zeolite,  Quartz | 230-240 | 4.53 | 4.21 | 4.20 | 17.68 |
| Calcite | 230-240 | 3.88 | 1.08 | 3.34 | 3.61 |
| Quartz | 230-240 | 3.36 | 1.63 | 2.90 | 4.73 |
| Calcite | 230-240 | 3.05 | 1.48 | 49.37 | 73.07 |
| Calcite | 230-240 | 2.50 | 1.96 | 6.36 | 12.47 |
| Calcite | 230-240 | 2.29 | 1.38 | 13.79 | 19.03 |
| Calcite | 230-240 | 1.92 | 1.65 | 8.64 | 14.26 |
| Calcite | 230-240 | 1.88 | 1.13 | 11.49 | 12.98 |
| Calcite | 230-240 | 1.61 | 0.98 | 5.06 | 4.96 |
| Smectite,  Zeolite | 220-230 | 6.00 | 1.99 | 0.81 | 1.61 |
| Smectite,  Zeolite | 220-230 | 4.53 | 2.53 | 3.03 | 7.67 |
| Calcite | 220-230 | 3.88 | 0.98 | 5.97 | 5.85 |
| Quartz | 220-230 | 3.36 | 1.91 | 1.87 | 3.57 |
| Calcite | 220-230 | 3.05 | 1.51 | 47.21 | 71.29 |
| Calcite | 220-230 | 2.50 | 1.81 | 5.58 | 10.10 |
| Calcite | 220-230 | 2.29 | 1.87 | 9.92 | 18.55 |
| Calcite | 220-230 | 1.92 | 2.06 | 30.13 | 62.07 |
| Calcite | 220-230 | 1.88 | 1.25 | 11.93 | 14.91 |
| Calcite | 220-230 | 1.61 | 1.00 | 10.04 | 10.04 |
| Smectite,  Zeolite | 210-220 | 6.00 | 1.59 | 0.94 | 1.49 |
| Smectite,  Zeolite,  Quartz | 210-220 | 4.53 | 1.73 | 3.08 | 5.33 |
| Calcite | 210-220 | 3.88 | 1.08 | 8.48 | 9.16 |
| Quartz | 210-220 | 3.36 | 1.03 | 3.41 | 3.51 |
| Calcite | 210-220 | 3.05 | 2.60 | 40.80 | 106.08 |
| Calcite | 210-220 | 2.50 | 1.30 | 8.36 | 10.87 |
| Calcite | 210-220 | 2.29 | 1.95 | 10.95 | 21.35 |
| Calcite | 210-220 | 1.92 | 2.25 | 11.25 | 25.31 |
| Calcite | 210-220 | 1.88 | 2.24 | 12.75 | 28.56 |
| Calcite | 210-220 | 1.61 | 1.51 | 6.07 | 9.17 |
| Calcite | 200-210 | 3.05 | 2.61 | 19.82 | 51.73 |
| Calcite | 200-210 | 2.50 | 2.09 | 6.09 | 12.73 |
| Calcite | 200-210 | 2.29 | 2.19 | 8.02 | 17.56 |
| Calcite | 200-210 | 1.92 | 2.31 | 5.30 | 12.24 |
| Calcite | 200-210 | 1.88 | 2.64 | 6.52 | 17.21 |
| Calcite | 200-210 | 1.61 | 1.80 | 5.49 | 9.88 |
| Smectite,  Zeolite,  Quartz | 190-200 | 4.53 | 3.80 | 5.08 | 19.30 |
| Calcite | 190-200 | 3.88 | 1.34 | 4.30 | 5.76 |
| Quartz | 190-200 | 3.36 | 1.58 | 6.48 | 10.24 |
| Calcite | 190-200 | 3.05 | 2.26 | 63.74 | 144.05 |
| Calcite | 190-200 | 2.5 | 1.71 | 10.08 | 17.24 |
| Calcite | 190-200 | 2.29 | 1.56 | 12.33 | 19.23 |
| Calcite | 190-200 | 1.92 | 2.31 | 10.27 | 23.72 |
| Calcite | 190-200 | 1.88 | 1.77 | 11.02 | 19.51 |
| Calcite | 190-200 | 1.61 | 1.34 | 5.57 | 7.46 |
| Smectite,  Zeolite, Quartz | 180-190 | 4.53 | 3.10 | 5.20 | 16.12 |
| Calcite | 180-190 | 3.88 | 2.08 | 9.80 | 20.38 |
| Quartz | 180-190 | 3.36 | 1.13 | 8.13 | 9.19 |
| Calcite | 180-190 | 3.05 | 2.38 | 28.53 | 67.90 |
| Calcite | 180-190 | 2.50 | 0.96 | 20.17 | 19.36 |
| Calcite | 180-190 | 2.29 | 1.53 | 23.36 | 35.74 |
| Calcite | 180-190 | 1.92 | 1.57 | 15.62 | 24.52 |
| Calcite | 180-190 | 1.88 | 1.41 | 19.31 | 27.23 |
| Calcite | 180-190 | 1.61 | 1.40 | 10.16 | 14.22 |
| Smectite,  Zeolite,  Quartz | 170-180 | 4.53 | 2.85 | 3.74 | 10.66 |
| Calcite | 170-180 | 3.88 | 0.93 | 10.60 | 9.86 |
| Quartz | 170-180 | 3.36 | 1.86 | 4.25 | 7.91 |
| Calcite | 170-180 | 3.05 | 2.68 | 31.36 | 84.04 |
| Calcite | 170-180 | 2.50 | 1.16 | 25.58 | 29.67 |
| Calcite | 170-180 | 2.29 | 1.33 | 28.97 | 38.53 |
| Calcite | 170-180 | 1.92 | 1.44 | 11.27 | 16.23 |
| Calcite | 170-180 | 1.88 | 1.60 | 17.74 | 28.38 |
| Calcite | 170-180 | 1.61 | 1.28 | 14.39 | 18.42 |
| Smectite,  Zeolite,  Quartz | 160-170 | 4.53 | 2.93 | 2.66 | 7.79 |
| Calcite | 160-170 | 3.88 | 0.83 | 4.22 | 3.50 |
| Quartz | 160-170 | 3.36 | 1.12 | 8.26 | 9.25 |
| Calcite | 160-170 | 2.50 | 1.13 | 16.02 | 18.10 |
| Calcite | 160-170 | 2.29 | 1.19 | 16.13 | 19.19 |
| Calcite | 160-170 | 1.92 | 1.02 | 6.76 | 6.90 |
| Calcite | 160-170 | 1.88 | 1.30 | 12.47 | 16.21 |
| Calcite | 160-170 | 1.61 | 1.48 | 9.84 | 14.56 |
| Smectite,  Zeolite,  Quartz | 310-300 | 4.41 | 1.18 | 1.84 | 2.17 |
| Calcite | 310-300 | 3.83 | 0.69 | 2.79 | 1.93 |
| Calcite | 310-300 | 3.01 | 1.60 | 15.60 | 24.96 |
| Calcite | 310-300 | 2.49 | 0.82 | 1.64 | 1.34 |
| Calcite | 310-300 | 2.27 | 1.33 | 4.37 | 5.81 |
| Calcite | 310-300 | 1.90 | 1.15 | 3.75 | 4.31 |
| Calcite | 310-300 | 1.87 | 0.96 | 6.45 | 6.19 |
| Calcite | 310-300 | 1.60 | 1.13 | 4.40 | 4.97 |
| Smectite,  Zeolite,  Quartz | 290-300 | 4.41 | 4.04 | 5.40 | 21.82 |
| Calcite | 290-300 | 3.83 | 1.97 | 4.10 | 8.08 |
| Quartz | 290-300 | 3.31 | 1.42 | 6.78 | 9.63 |
| Calcite | 290-300 | 3.01 | 1.92 | 31.61 | 60.69 |
| Calcite | 290-300 | 2.49 | 1.56 | 18.95 | 29.56 |
| Calcite | 290-300 | 2.27 | 1.71 | 14.30 | 24.45 |
| Calcite | 290-300 | 1.90 | 1.98 | 10.24 | 20.28 |
| Calcite | 290-300 | 1.87 | 1.18 | 14.06 | 16.59 |
| Calcite | 290-300 | 1.6 | 1.12 | 6.04 | 6.76 |
| Calcite | 280-290 | 3.83 | 1.38 | 4.58 | 6.32 |
| Quartz | 280-290 | 3.31 | 1.56 | 7.29 | 11.37 |
| Calcite | 280-290 | 3.01 | 2.79 | 63.81 | 178.03 |
| Calcite | 280-290 | 2.49 | 1.34 | 9.81 | 13.15 |
| Calcite | 280-290 | 2.27 | 1.26 | 15.52 | 19.56 |
| Calcite | 280-290 | 1.90 | 1.26 | 9.65 | 12.16 |
| Calcite | 280-290 | 1.87 | 1.25 | 13.81 | 17.26 |
| Calcite | 280-290 | 1.60 | 0.99 | 6.67 | 6.60 |
| Calcite | 270-280 | 3.83 | 1.18 | 5.17 | 6.10 |
| Quartz | 270-280 | 3.31 | 1.17 | 5.17 | 6.05 |
| Calcite | 270-280 | 3.01 | 2.82 | 54.65 | 154.11 |
| Calcite | 270-280 | 2.49 | 1.19 | 8.73 | 10.39 |
| Calcite | 270-280 | 2.27 | 1.08 | 11.89 | 12.84 |
| Calcite | 270-280 | 1.90 | 1.18 | 9.99 | 11.79 |
| Calcite | 270-280 | 1.87 | 1.32 | 10.56 | 13.94 |
| Calcite | 270-280 | 1.60 | 1.24 | 6.41 | 7.95 |
| Smectite,  Zeolite,  Quartz | 260-270 | 4.41 | 0.86 | 1.92 | 1.65 |
| Calcite | 260-270 | 3.01 | 0.76 | 28.91 | 21.97 |
| Calcite | 260-270 | 2.49 | 0.92 | 5.68 | 5.23 |
| Calcite | 260-270 | 2.27 | 1.53 | 10.95 | 16.75 |
| Calcite | 260-270 | 1.87 | 1.03 | 8.94 | 9.21 |
| Calcite | 260-270 | 1.60 | 1.16 | 5.61 | 6.51 |
| Smectite,  Zeolite,  Quartz | 250-260 | 4.41 | 2.09 | 1.09 | 2.28 |
| Calcite | 250-260 | 3.01 | 1.46 | 12.69 | 18.53 |
| Calcite | 250-260 | 2.49 | 1.97 | 2.66 | 5.24 |
| Calcite | 250-260 | 2.27 | 1.64 | 6.90 | 11.32 |
| Calcite | 250-260 | 1.9 | 1.29 | 2.49 | 3.21 |
| Calcite | 250-260 | 1.87 | 1.27 | 2.09 | 2.65 |
| Calcite | 250-260 | 1.60 | 0.87 | 2.33 | 2.03 |
| Calcite | 240-250 | 3.83 | 0.80 | 2.19 | 1.75 |
| Quartz | 240-250 | 3.31 | 1.02 | 6.07 | 6.19 |
| Calcite | 240-250 | 3.01 | 2.40 | 24.49 | 58.78 |
| Calcite | 240-250 | 2.49 | 1.22 | 4.89 | 5.97 |
| Calcite | 240-250 | 2.27 | 1.21 | 9.97 | 12.06 |
| Calcite | 240-250 | 1.87 | 1.66 | 12.29 | 20.40 |
| Calcite | 240-250 | 1.60 | 1.16 | 2.57 | 2.98 |

**
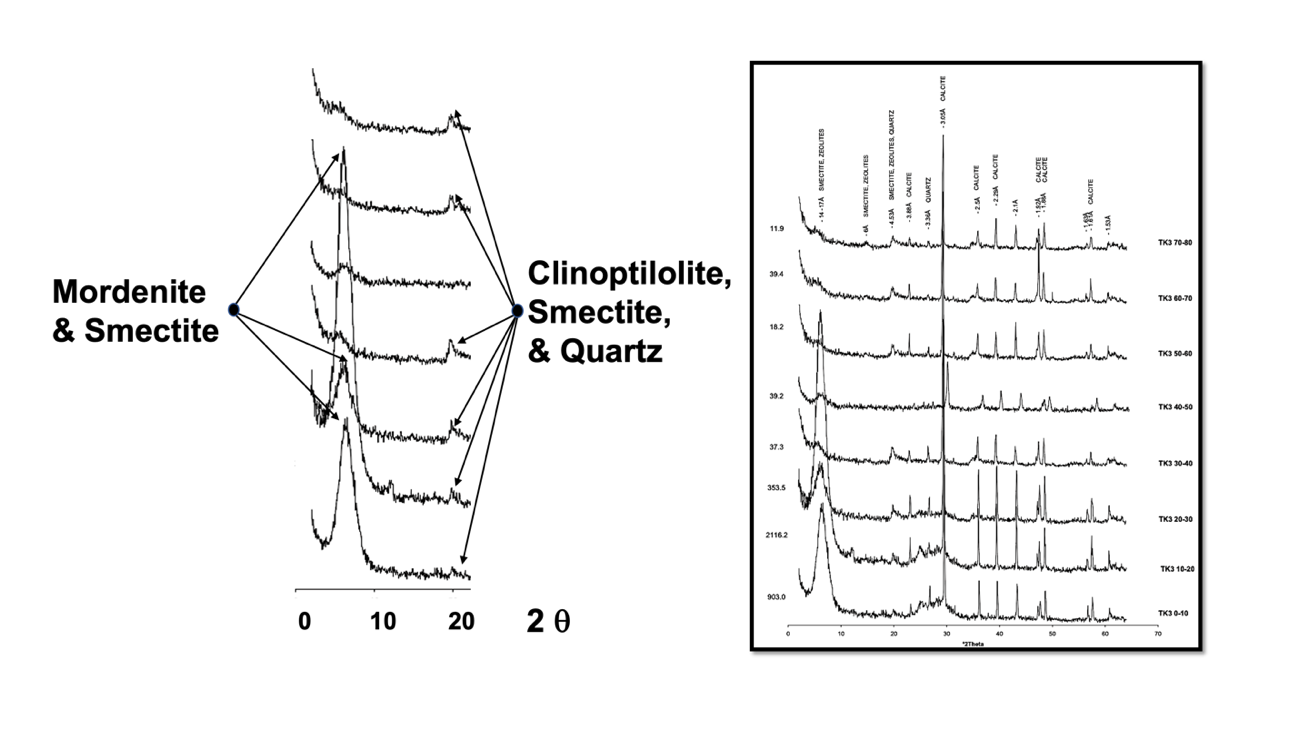
**

**fig. S10. Composite X-ray diffractograms (N=8) of the mineralogy of sediments**

**extracted from the Corriental reservoir.** Kenneth Barnett Tankersley used Microsoft PowerPoint for Mac Version 16.41 ([www.microsoft.com](http://www.microsoft.com)) to create this figure.

**
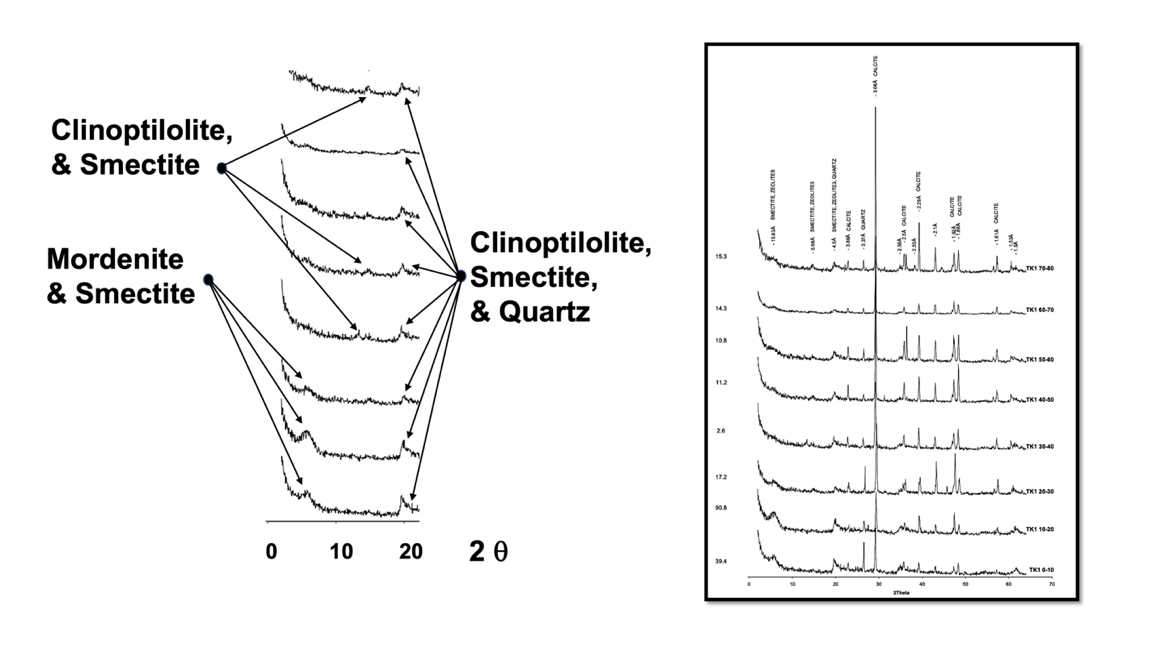
**

**fig. S11. Composite X-ray diffractograms (N=8) of the mineralogy of sediments extracted from the Corriental reservoir.** Kenneth Barnett Tankersley used Microsoft PowerPoint for Mac Version 16.41 ([www.microsoft.com](http://www.microsoft.com)) to create this figure.


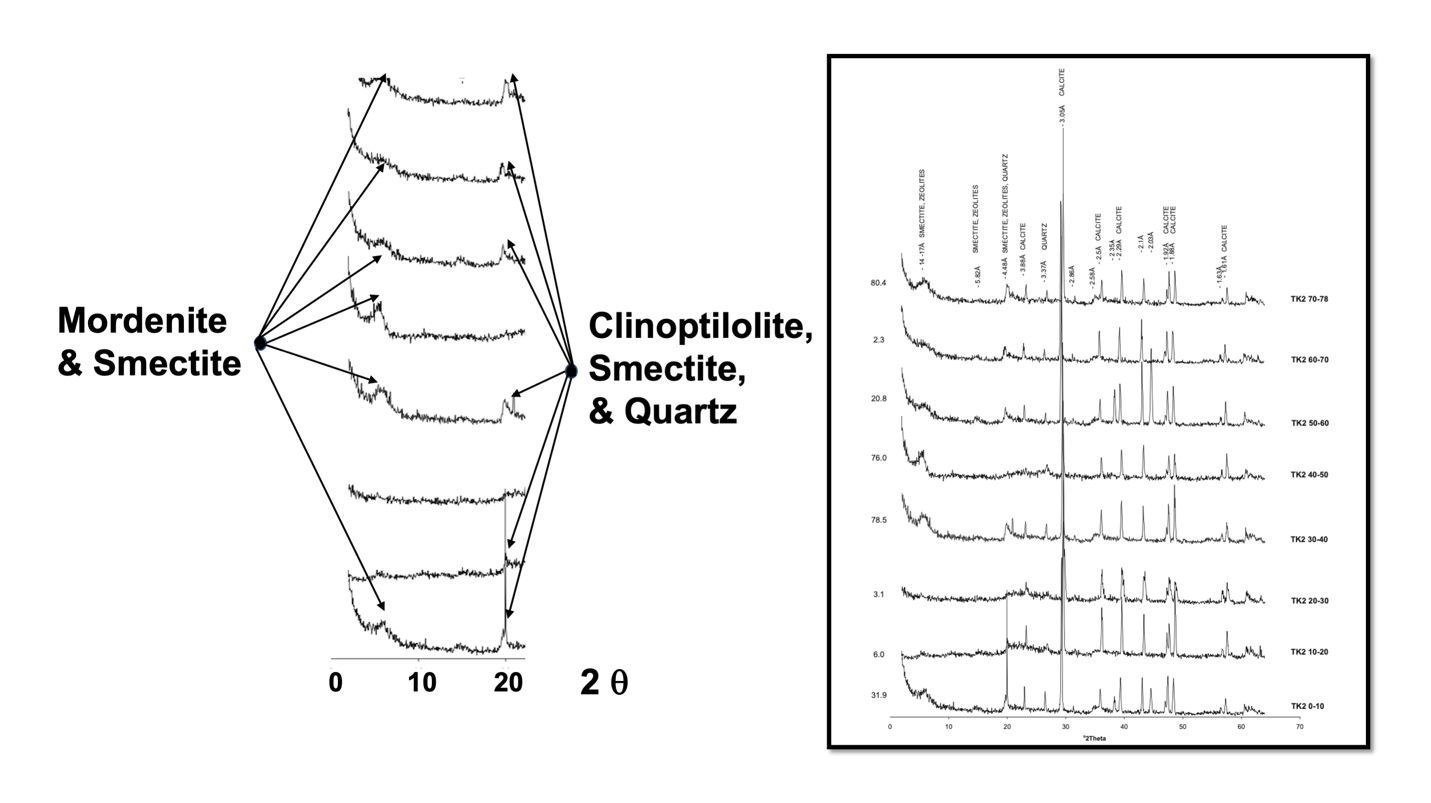


**fig. S12. Composite X-ray diffractograms (N=8) of the mineralogy of sediments extracted from the Corriental reservoir.** Kenneth Barnett Tankersley used Microsoft PowerPoint for Mac Version 16.41 ([www.microsoft.com](http://www.microsoft.com)) to create this figure.


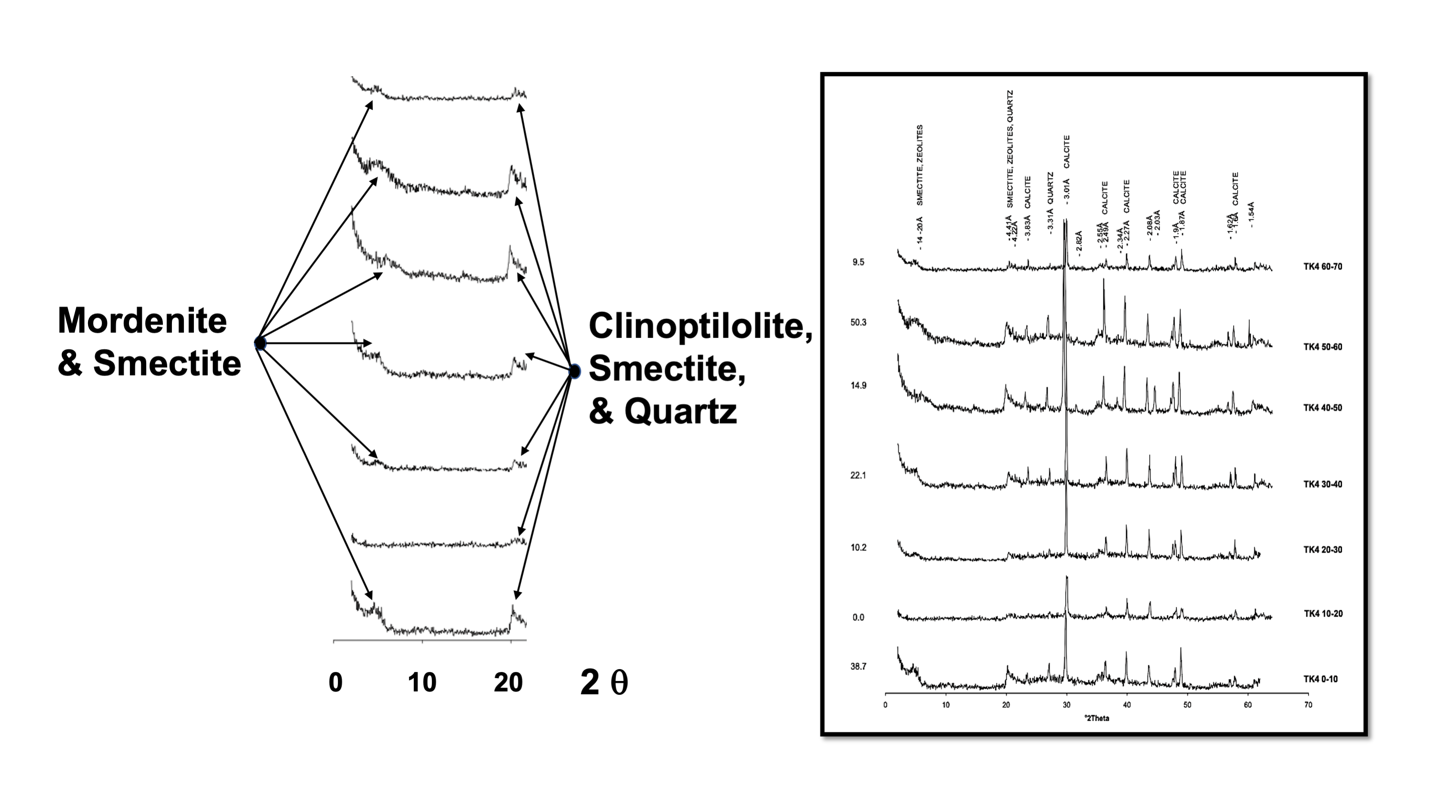


**fig. S13. Composite X-ray diffractograms (N=8) of the mineralogy of sediments extracted from the Corriental reservoir.** Kenneth Barnett Tankersley used Microsoft PowerPoint for Mac Version 16.41 ([www.microsoft.com](http://www.microsoft.com)) to create this figure.

1. **Archaeological investigations of ancient Maya reservoirs and water cleanliness**

Within the Maya Lowlands lies an irregularly-shaped physiographic province known as the Elevated Interior Region (EIR), a karst area characterized by an acute lack of perennial surface water and almost no access to groundwater (S13). Given the 5-month-long dry season within the regional climate, year-round occupation by large numbers of Maya people and urbanization was dependent on the ability to capture and store large quantities and rain water.

*5.1 Early archaeological investigations*

One of the first archaeological explorations of the northern part of the EIR by John Lloyd Stephens and artist companion Frederick Catherwood noted: “Among the wonders unfolded by the discovery of these ruined cities, what made the strongest impression on our minds was the fact that their immense population existed in a region so scantily supplied with water (S14).” Catherwood illustrated several ancient reservoirs, including a hypothetical cross-section of one that had been cleaned out by an agricultural estate owner and included a number of chambers and wells constructed in the floor of reservoir. The 19^th^ century reuse of ancient reservoirs was a not uncommon practice in the northern EIR (S15) Early explorers of the EIR, both archaeological and economic (e.g. chicle gatherers) were typically utterly dependent on obtaining water during the dry season from pools within ancient Maya reservoirs and acutely aware of their association with ruined settlements (S16, S17, S18, S19).

*5.2 Twentieth century*

The first modern and *bona fide* archaeological excavations of ancient Maya reservoirs took place in the 1950s and 1960s as part of the University of Pennsylvania Tikal Project (see section S1). Unfortunately, reports on these excavations were never completed or published (S3).

Beginning in the late 1970s, reservoirs have received increasing attention in archaeological investigations in the EIR. Investigated sites have included El Mirador, Guatemala (S20, S21), Edzna, Mexico (S22), Uxmal, Mexico and other Puuc sites (S13, S23), Kinal, Guatemala (S24), La Milpa and other sites in the Programme for Belize (S25, S26, S27), Calakmul, Mexico (S28, S29), Tamarindito, Guatemala (S30); Zacatal, near Nakbe, Guatemala (S31), San Bartolo, Xultun, and nearby sites in Guatemala (S32, S33), Xuch, Mexico (S34), Xcoch, Mexico (S35), Oxpemul, Mexico (S36), Uxul, Mexico (S37), and El Zotz, Guatemala (S38). Numerous studies have also investigated the technology and capacity of household scale water catchment and storages features: chultuns or cisterns in the northern EIR (S29, S30, S31, S32) and open-air tanks in the southern EIR (S33, S34). Ongoing investigations at Yaxnohcah and surrounding areas, Mexico have included excavations and coring in eleven reservoirs and many household tanks (S35, S36). The various investigations listed above have been conducted for a variety of reasons including gaining an understanding of ancient Maya water management, but also to obtain geochemical and paleoecological proxy data that have been used in paleoenvironmental reconstructions and modeling.

*5.3 Our current understanding of ancient Maya water cleanliness*

In the course of these investigations, data has been recovered on techniques that the ancient Maya employed to improve or maintain water purity. Given that the ancient Maya would not have had the capacity to visualize or test for microscopic and chemical impurities, we can assume that transparency, color, taste, and smell were used to gauge water quality. Since many reservoir catchments included areas of open soil, sediment load and associated turbidity in runoff water were needed to be dealt with. Some reservoirs fed by fluvial systems included siltation or settling tanks, open pools created in stream beds before reservoir ingresses that functioned to slow incoming water allowing sediment to precipitate. Examples of siltation tanks are known from Kinal (S24), Los Loros, near San Bartolo (S33), Uxul (S37), and El Zotz (S38). At Uxul one of the reservoirs also included a “filtration wall” – a wall of heavy laid stones that presumably reduced the amount of clastic sediment entering the reservoir. Notably, remains of this wall were not visible prior to excavation and such features may be more commonplace. It is distinctly possible that the sand filters at Tikal were retained behind similar walls. A large number of Maya reservoirs were constructed within bajos, but without apparent inlets suggesting that they were filled solely by rainfall, which was another means of maintaining cleaner water; berm walls built around these reservoirs not only increased their capacity, but also functioned to exclude turbid water from the surrounding bajos in the rainy season (S39).

Within some reservoirs, *buk’té’ob* (Yukatek Mayan, singular *buk’té*) or filtration wells were constructed; these wells feature dry-laid stone walls surrounding openings of varying depth and width. In clay-bottomed reservoirs, or those with significant accumulated sediment into which the well was sunk, through-flow of water from surrounding saturated sediments could be accessed to obtain a final water yield from the drying reservoir. Known examples of *buk’té’ob*occur at Uxmal (S24), Ichpich, Halal, and Tzeme Akal in the Puuc region (S15, S16, S40), Lagunita Elusiva and La Milpa, Belize (S28, S41), and Yaxnohcah, Mexico.

Classic period iconography and inscriptions indicate that Maya rulers associated themselves with pure water and with water lilies. While water lilies (*Nymphaea* spp.) are rarely found in ancient reservoirs that still hold water today, pollen recovered from sediments in a few reservoirs do contain lily pollen, supporting the idea that these plants were propagated at least in some reservoirs (S42). Lilies would have the beneficial properties of slowing evaporation off of water surfaces, as well as biologically filtering some toxins that may have been present in reservoirs (S43). More environmental archaeological investigation on this question is needed.

To date, excavations have been conducted in only a few dozen of the many thousands of ancient reservoirs in the EIR, and within many of these excavations have been limited to a single test pit. Hence, in the future we are likely to learn a great deal more about ancient Maya water capture and storage technology, possibly including the more extensive use of sand filtration (S44, S45).

1. **Ancient Maya potable water: clarity vs. water purity**

*6.1 Terminology*

Purified refers to potable water, which has been filtered to remove impurities. Potable refers to water, which is suitable for drinking without getting sick. Clear refers to water, which is completely transparent and colorless. Equating clear and potable water is a common misconception because water can be clear, but not potable.

*6.2 Contaminants*

Contaminants in ancient Maya reservoir and well water likely included harmful microbial pathogens, soluble organics, and heavy metal leachates. The origins of these toxins were likely anthropogenic (domestic urban and rural waste and human sewage), biologic (algae, bacteria, parasites, protozoa, viruses, and worms), and geologic (arsenic—orpiment and realgar, lead—cerussite and galena, and mercury—cinnabar). These toxins probably presented the ancient Maya with both short and long-term diseases (S46). The adverse effects of drinking contaminated water would have resulted malnutrition and diarrhea and children would have been especially vulnerable (S47). Central city reservoirs would have been especially difficult to keep clean depending on what kinds of sanitation options were, or were not, in place

The ancient Maya likely used the color, smell, and taste of the water to evaluate whether or not water was potable. However, these techniques would have been unreliable. Odorless, clear, sweet tasting water can be contaminated with pathogens. Similarly, water that is colored or cloudy (from air bubbles, clay, iron, sand, silt, tannin) or smells or tastes bad (from sulfur) may be potable. For example, the clay mineral kaolinite will cause water to look opaque and milky. However, kaolinite is not toxic. Indeed, it is an ancient Indigenous medicine used for the treatment of cholera, enteritis, and dysentery (S48). While kaolinite has no antibacterial properties nor is it a valid treatment for infectious diseases, it has been used effectively for symptoms associated with contaminated water such as diarrhea and an upset stomach (S48). Kaolinite has been identified at Tikal in the Aguada de Terminos, Corriental Arroyo, and the Corriental Bajo.

*6.3 Karst landscape*

The city of Tikal was built on a karst landscape. Karst topography results from the dissolution of underlying bedded and jointed limestone bedrock. These horizontal and vertical fractures provide a direct channel between the land’s surface and the groundwater system. Karst aquifers are vulnerable to anthropogenic, biologic, and geologic toxins because of rapid hydraulic responses, focused channelized flow regimes, and fast groundwater flow and transport in conduits (S49). Groundwater rapidly recharges entering karst aquifers through sinkholes and resurfacing from springs. Water moves quickly over great distances (1 km/day) without minimal or no form of natural filtration processes (S49). Throughout the Elevated Interior Region, including Tikal, the permanent groundwater table lies between one to three hundred meters below the ground surface and generally out of the reach of the ancient Maya. Perched aquifers sporadically occur at shallower depths, including that which feeds the Tikal Temple reservoir spring. The depth of the Bajo de Azúcar aquifer is not well understood. It was discharging in a scarp some 30-40 meters below the overlying ground surface where observed.

*6.4 Water purification*

Clastic water filters constructed from boulders, cobbles, gravel, sand, or some combination of clasts, can be an effective means to physically clarify water. Clastic filters can provide a quick and effective means to remove suspended particulate matter such as clay and silt from water. Sand water filters can completely eliminate suspended solids. However, the ability of sand filters to remove pathogens depends upon the thickness of the sand, the temperature of the water, and the concentration of suspended particulate matter (S50). For sand filters to be useful, the algae count, temperature, and turbidity all must be low (S50, S51).

The ancient Maya of Tikal had clastic water filtration technology, an organized and planned water management infrastructure, and access to the raw materials needed to create an efficient purification system. Zeolite is the most effective natural raw material known to successfully remove harmful microbial pathogens, soluble organics, and heavy metal leachates (S51). When zeolite was added to sand filters, the ancient Maya of Tikal created the most effective water purification system in prehistory.

**7. Inscriptions sinkhole control**

We use the “Inscriptions Sinkhole” as a control in this article, arguing that it was more likely a natural karst sinkhole, though perhaps modified by the ancient Maya including enclosing it within a berm wall. This feature was identified as a reservoir, named, and mapped by the University of Pennsylvania Tikal Project. Superficially, this circular depression resembles a number of other reservoirs at Tikal, including Corriental and Perdido. However, these three features differ in some important respects. Corriental and Perdido are situated in significant drainages with catchments that would have funneled significant surface flow into their central depressions (Fig. 1); the Inscriptions sinkhole does not. Corriental and Perdido have surface outlets allowing excess water to be discharged; Inscriptions does not. The interior “tank” of Inscriptions is significantly deeper than either Corriental or Perdido and resembles a karst sinkhole or *rejollada*. Although the University of Cincinnati Tikal Project did not excavate within Inscriptions in 2009 or 2010, we did obtain two solid-sediment percussion cores from its floor. The resulting core profiles are different than those of the excavated and cored reservoirs at Tikal showing no stratified sediments, resembling more closely the Vertisols (soils that “self-invert” due to expansion and contraction of clay) characteristic of natural depressions in the southern and central Maya Lowlands. Four radiocarbon dates obtained from soil organic matter at depths between 50 and 130 cm in one of the cores ranged from 3,000 to 13,700 cal yr. BP with considerable inversion of dates, a finding also indicative of a Vertisol. The vegetation within Inscriptions is also quite different than found in any Tikal’s reservoirs and included two tree species, *Hymenaea courbaril* and *Gliricidia sepium*, the latter known commonly as “Madre de Cacao.” Both trees are cacao (*Theobroma cacao*) symbionts and are often cultivated as shade trees in cacao plantations. We have suggested elsewhere (S11 [see note #8]) that the preponderance of evidence indicates that “Inscriptions Reservoir” was likely not a reservoir at all and may have been used as a royal cacao grove. The climate at Tikal is not suitable for cacao, but the sinkhole would have provided ample soil humidity for its growth. Similar sinkholes are known to have been used for cacao growth as far north as Yucatan State in the 16^th^ century CE and were the exclusive domain of royal or other elite families (S52). Notably, such groves were often surrounded by walls as is Inscriptions (S52).

**8. Bajo de Azúcar**

The Bajo de Azúcar (“Sugar Bajo”) is a sprawling depression coving some 600 km^2^ in the northeastern part of the Peten Distict of Guatemala, the southern edge of which lies between 30 and 35 km NNE of Tikal. The bajo was created by a combination of normal faulting and limestone dissolution leaving steep scarps along portions of its margins. In 2007 and 2008 teams from the Proyecto Arqueológico San Bartolo ventured into the Bajo de Azúcar to examine and excavate linear features visible on satellite imagery. Near the southern end of the bajo they excavated a linear feature (verified as an ancient Maya canal) at the base of a steep slope forming the western margin of an island of high ground within the bajo. Two excavations exposed a layer of porous stone formed chiefly of sand-sized quartz crystals, which was actively discharging clear water; samples of the stone were collected at the time and brought back to Cincinnati for analysis. The stone was thought at first to be a highly weathered sandstone, but, as reported in the text, the absence of detrital quartz and the presence of macrocrystalline quartz and zeolite in this rock makes its identification as a very weathered volcanic tuff more likely. Neither tuff nor sandstone have been previously reported in the Late Cretaceous strata of this part of the Peten District, but it is very poorly known. Residents of the town of Uaxactun who are familiar with parts of the Bajo de Azúcar, report that other springs discharging from what they refer to as “sandstone” aquifers are known along other edges of the bajo (e.g., at the poorly known ancient Maya site today called Manantial, or “Big Spring” (S53). The origin of the name “Sugar Bajo” is unknown. However, it has been suggested that the sand-sized quartz crystals weathering from its margins and accumulating at a fluvial choke point at the north end of the bajo may be the source of the place name given that sand-sized quartz crystals are extremely rare in this area of the Maya Lowlands. Notably, the aquifers along the margins of the bajo are also locally known for their clear, clean, and “sweet” water, which may also explain the place name.

Although the ancient Maya would have not been aware of the presence of the co-occurrence of crystalline quartz and zeolites in the weathering tuffs around the Bajo de Azúcar, they would certainly have observed the apparent cleanliness of the water discharging from the associated aquifer. This observation may explain why the Maya of Tikal went to the trouble of transporting these minerals, or perhaps blocks of the weathering coarsely crystalline tuff, to their city for inclusion in their water system.

**9. Scheme of the ancient water purification system at Tikal.**


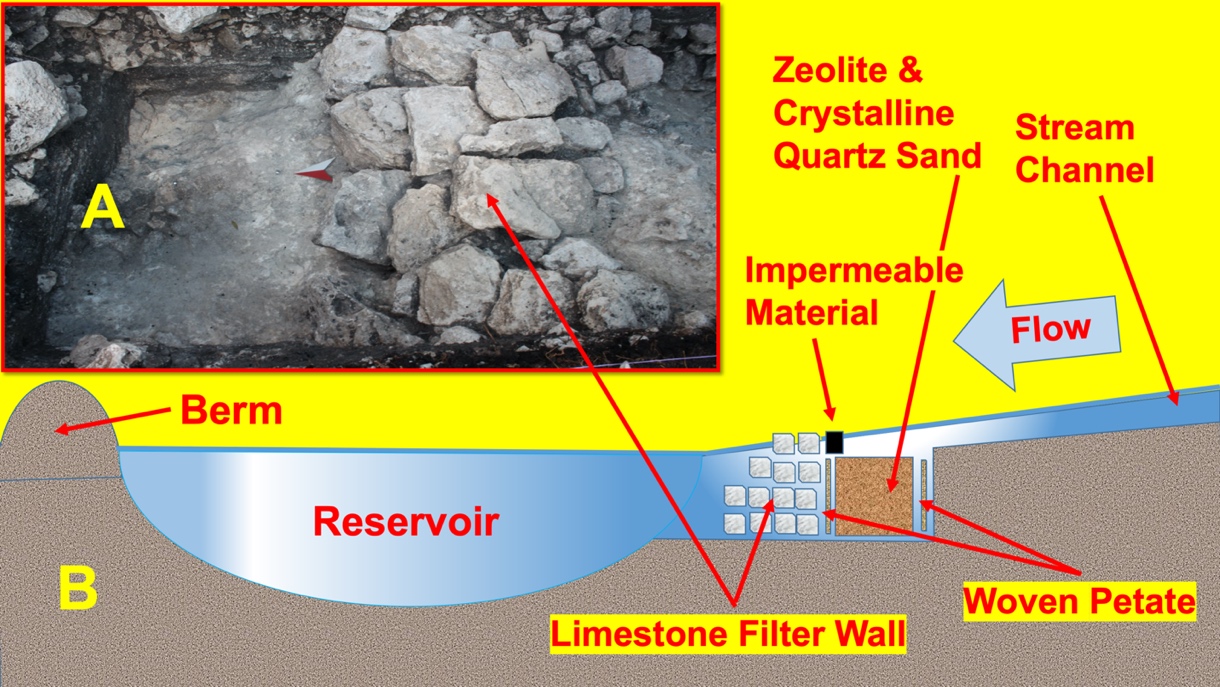


**fig. S13. Diagrammatic scheme of the ancient water purification system at Tikal.** (A) A dry limestone structure at the Aguada Orriental reservoir at Uxul (S37, S54, fig. 6.32). (B) A cross-section of the macro-crystalline quartz crystal sand and zeolite filtration system positioned upstream of the reservoir ingress. Kenneth Barnett Tankersley used Microsoft PowerPoint for Mac Version 16.41 ([www.microsoft.com](http://www.microsoft.com)) to create this figure.

The Tikal water filtration system was likely contained by dry laid stone walls, which supported macrocrystalline sand-sized quartz further constrained by woven petates (mats of woven reeds, palm leaves, or other fibers) or other porous materials. A stone filtration wall was discovered *in situ* at an ingress of the Aguada Orriental reservoir at Uxul (S37, S54). Behind the wall at Uxul was a depression carved into bedrock which was interpretted as a settling/siltation tank, but which could have at one time contained a filter. The walls of the Corriental filtration system may have been bigger because the velocity of the inflowing arroyo was likely stronger (i.e., a bigger catchment with a steeper gradient).

**10. Provenance Analysis of Anthropogenic Mercury**

Mercury (Hg) enriched sediment samples were collected from Tikal reservoir contexts, which do not have local bedrock cinnabar mineralization. Mercury enrichment of the reservoir sediments occurs in the clay-sized fraction because of the high adsorption capacity of smectite particles for Hg (S55). Smectite is a volcanogenic clay, which is abundant in the reservoir sediments of Tikal. In order to determine the source of Hg, major and trace element analysis of the Hg-rich reservoir sediments was conducted using energy dispersive-X-ray fluorescence spectrometry (ED-XRF) (S55). The results of this analysis are presented in table S7.

In Guatemala, Hg occurs naturally in the form of the mineral cinnabar (HgS) (S56). The closest source of cinnabar to Tikal is the Jurassic to Early Cretaceous age Todos Santos Formation, which is located in southwestern Guatemala. The formation consists of limestone, polymictic conglomerate, sandstone, mudstone, volcanic and volcaniclastic rocks (S57, S58). The positive anomalies of the major elements phosphorus (P_2_O_5,_ 0.62-0.76%) and potassium (K_2_O, 0.79-0.92%), and the trace element strontium (Sr, 130.04-275.25 ppm) in the clay fraction of Tikal’s Hg-rich reservoir sediments is consistent with what we would expect to find for cinnabar from the Todos Santos Formation (Godínez-Urban et al., 2011). The positive anomaly for calcium (CaO, 61.11-81.60) is not unexpected as calcite (CaCO_3_) is a common associate mineral of cinnabar (S59). The positive Ca and Sr anomalies are noteworthy as they are elements, which are accumulated by cyanobacterium (S60).

**table S7. ED-XRF analysis of Tikal’s Hg-rich Reservoir sediments.**

| **Trace Elements (ppm)** | | | | | | | | | | | | | | | | | | | | | | | | | | | | | |
| --- | --- | --- | --- | --- | --- | --- | --- | --- | --- | --- | --- | --- | --- | --- | --- | --- | --- | --- | --- | --- | --- | --- | --- | --- | --- | --- | --- | --- | --- |
| **Reservoir Sample** | **V** | | | **Cr** | | | **Co** | | | **Ni** | | | **Cu** | | | **Zn** | | | **Rb** | | | **Sr** | | | **Y** | | **Zr** | | |
| Palace | 0.00 | | | 19.13 | | | 0.00 | | | 14.31 | | | 27.18 | | | 55.80 | | | 43.32 | | | 135.76 | | | 23.72 | | 116.71 | | |
| Palace | 28.26 | | | 23.14 | | | 18.64 | | | 8.90 | | | 15.15 | | | 27.15 | | | 24.29 | | | 130.04 | | | 10.54 | | 62.82 | | |
| Palace | 0.00 | | | 20.22 | | | 0.00 | | | 15.29 | | | 11.12 | | | 24.19 | | | 19.02 | | | 179.66 | | | 12.23 | | 71.51 | | |
| Palace | 0.00 | | | 46.15 | | | 0.00 | | | 12.54 | | | 13.50 | | | 24.86 | | | 15.98 | | | 136.60 | | | 12.55 | | 60.76 | | |
| Palace | 46.05 | | | 0.00 | | | 5.80 | | | 0.00 | | | 4.61 | | | 14.71 | | | 9.43 | | | 257.25 | | | 9.65 | | 38.03 | | |
| Palace  (Range) | 0-46 | | | 0-46 | | | 0-18 | | | 0-15 | | | 5-27 | | | 14-56 | | | 9-43 | | | 130-257 | | | 10-23 | | 38-117 | | |
| Corriental  (Range) | 32-46 | | | 31-38 | | | ND | | | 28-41 | | | ND | | | ND | | | 16-19 | | | 40-42 | | | 30-38 | | 110-146 | | |
| Perdido  (Range) | 22-27 | | | 76-87 | | | ND | | | 61-76 | | | ND | | | ND | | | 30-32 | | | 22-27 | | | 36-38 | | 158-214 | | |
| Perdido Pre-habitation  Control | 156.71 | | | 108.87 | | | 94.74 | | | 151.60 | | | 49.18 | | | 81.86 | | | 55.67 | | | 36.49 | | | 54.76 | | 374.60 | | |
| **Trace Elements (ppm)** | | | | | | | | | | | | | | | | | | | | | | | | | | | | | |
| **Reservoir Sample** | | **Nb** | **Mo** | | | **Cd** | | | **Sn** | | | **Sb** | | | **Te** | | | **Ba** | | | **La** | | | **Pb** | | **Th** | | | **Hg^1^** |
| Palace | | 0.00 | 0.00 | | | 0.00 | | | 1.84 | | | 2.46 | | | 0.75 | | | 211.52 | | | 69.97 | | | 16.70 | | 0.00 | | | BD |
| Palace | | 0.00 | 0.17 | | | 2.76 | | | 4.57 | | | 0.00 | | | 8.02 | | | 152.75 | | | 27.61 | | | 0.00 | | 10.44 | | | BD |
| Palace | | 1.67 | 0.93 | | | 0.49 | | | 5.21 | | | 0.43 | | | 5.75 | | | 112.18 | | | 38.51 | | | 12.93 | | 0.00 | | | BD |
| Palace | | 1.31 | 0.00 | | | 3.18 | | | 2.90 | | | 5.85 | | | 0.00 | | | 142.68 | | | 19.36 | | | 0.00 | | 0.00 | | | BD |
| Palace | | 0.00 | 0.00 | | | 0.00 | | | 4.43 | | | 0.00 | | | 1.70 | | | 107.87 | | | 57.87 | | | 9.24 | | 3.82 | | | BD |
| Palace  (Range) | | 0-2 | 0-1 | | | 0-3 | | | 2-5 | | | 0-6 | | | 0-8 | | | 108-212 | | | 19-70 | | | 0-17 | | 0-10 | | | BD |
| Corriental  (Range) | | 9-10 | ND | | | ND | | | ND | | | ND | | | ND | | | 132-350 | | | ND | | | ND | | ND | | | ND |
| Perdido  (Range) | | 14-16 | ND | | | ND | | | ND | | | ND | | | ND | | | 193-290 | | | ND | | | ND | | ND | | | ND |
| Perdido Pre-habitation  Control | | 24.07 | 0.00 | | | 2.63 | | | 5.93 | | | 0.68 | | | 0.00 | | | 649.47 | | | 51.09 | | | 50.53 | | 8.56 | | | BD |
| **Major Elements (%)** | | | | | | | | | | | | | | | | | | | | | | | | | | | | | |
| **Reservoir**  **Sample** | | **Na_2_O** | | **MgO** | **Al_2_O_3_** | | | **Si_2_O** | | | **P_2_O_5_** | | | **SO_2_** | | | **K_2_O** | | | **CaO** | | | **TiO_2_** | | **MnO** | | | **Fe_2_O_3_** | |
| Palace | | 0.68 | | 0.85 | 4.92 | | | 28.47 | | | 0.75 | | | 0.15 | | | 0.92 | | | 61.01 | | | 0.24 | | 0.07 | | | 1.86 | |
| Palace | | 1.03 | | 0.77 | 3.40 | | | 18.08 | | | 0.62 | | | 0.12 | | | 0.79 | | | 74.11 | | | 0.09 | | 0.03 | | | 0.90 | |
| Palace | | 1.49 | | 0.89 | 2.86 | | | 15.90 | | | 0.75 | | | 0.16 | | | 0.85 | | | 76.11 | | | 0.09 | | 0.03 | | | 0.82 | |
| Palace | | 0.40 | | 0.54 | 3.91 | | | 20.77 | | | 0.70 | | | 0.12 | | | 0.85 | | | 71.63 | | | 0.11 | | 0.03 | | | 0.90 | |
| Palace | | 1.81 | | 0.90 | 2.23 | | | 11.12 | | | 0.76 | | | 0.18 | | | 0.81 | | | 81.60 | | | 0.05 | | 0.02 | | | 0.47 | |
| Palace  (Range) | | 0-2 | | < 1 | 2-5 | | | 11-29 | | | < 1 | | | < 1 | | | < 1 | | | 61-82 | | | < 1 | | < 1 | | | 0-2 | |
| Corriental  (Range) | | 1.12-6.23 | | 0.13-0.15 | 10.8-14.9 | | | 28.4-40.1 | | | 0.12-0.16 | | | ND | | | 0.01-0.09 | | | 7.07-23.10 | | | 0.18-0.40 | | 0.09-0.25 | | | 2.41-4.20 | |
| Perdido  (Range) | | 0.04-0.05 | | 1.51-1.58 | 17.8-18.6 | | | 42.0-43.9 | | | 0.01 | | | ND | | | 0.17-0.18 | | | 7.63-9.68 | | | 0.40-0.45 | | 0.38-0.38 | | | 6.62-7.34 | |
| Perdido  Pre-habitation  Control | | 0.00 | | 1.06 | 13.67 | | | 57.13 | | | 0.01 | | | 0.04 | | | 0.46 | | | 14.60 | | | 1.23 | | 0.98 | | | 10.63 | |

ND = No Data (i.e., elemental data was collected).

BD = Below Detection (i.e., below the mercury contaminated soil standard [SRM 2710 - Montana Soil] certified to contain 32.6 ppm Hg). See fig, S13.


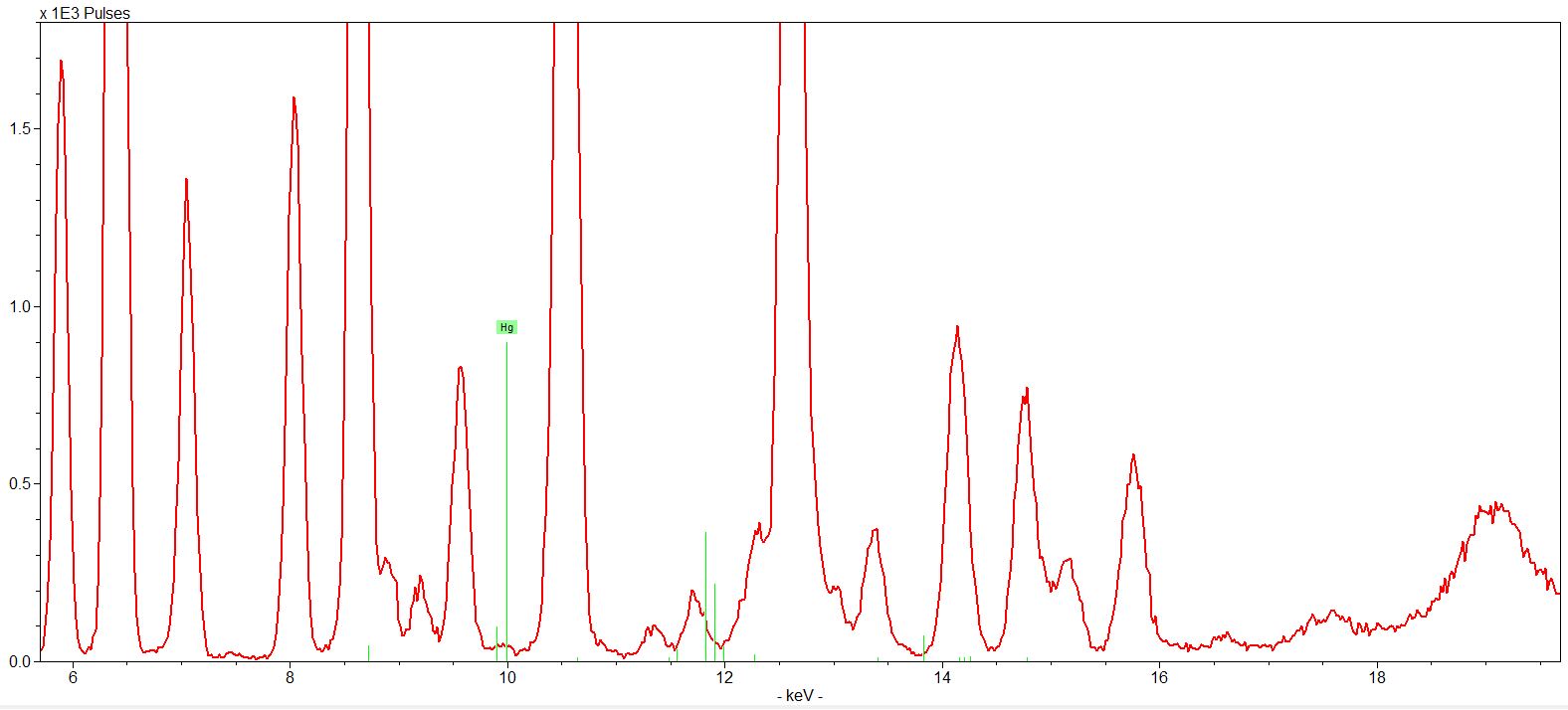


**fig. S14. Mercury contaminated soil standard (SRM 2710 - Montana Soil) certified to contain 32.6 ppm Hg.**Kenneth Barnett Tankersley used Microsoft PowerPoint for Mac Version 16.41 ([www.microsoft.com](http://www.microsoft.com)) to create this figure.

**11. Lidar-derived hillshade images with reservoir catchments.**

Lidar-derived hillshade images with reservoir catchments were produced by combining four data sets. The initial data consisted of the University of Pennsylvania maps of the Ruins of Tikal, El Peten, Guatemala (S61). These maps were used to locate the structures and reservoirs of central Tikal and provided elevation contour lines. The reservoir catchments were delineated by hand based on the contour lines on the University of Pennsylvania maps of Tikal. Christopher Carr converted the the University of Pennsylvania maps of Tikal and the hand-drawn reservoir and sinkhole catchments to an electronic format for use in Geographic Information System (GIS) software using georeferencing methods to create a GIS layer (S61, S62, S63).

The base maps are lidar-derived hillshade images created by Francisco Estrada-Belli, a principle of the PACUNAM Lidar initiative. The hillshade image was shared with the press by the Pacunam project (Pacunam.com) and published on-line (S. Christopher Carr georeferenced the hillshade images based on the georeferenced the University of Pennsylvania maps of Tikal. He then overlaid the GIS layer of the catchment areas on the georeferenced hillshade to make the base maps.


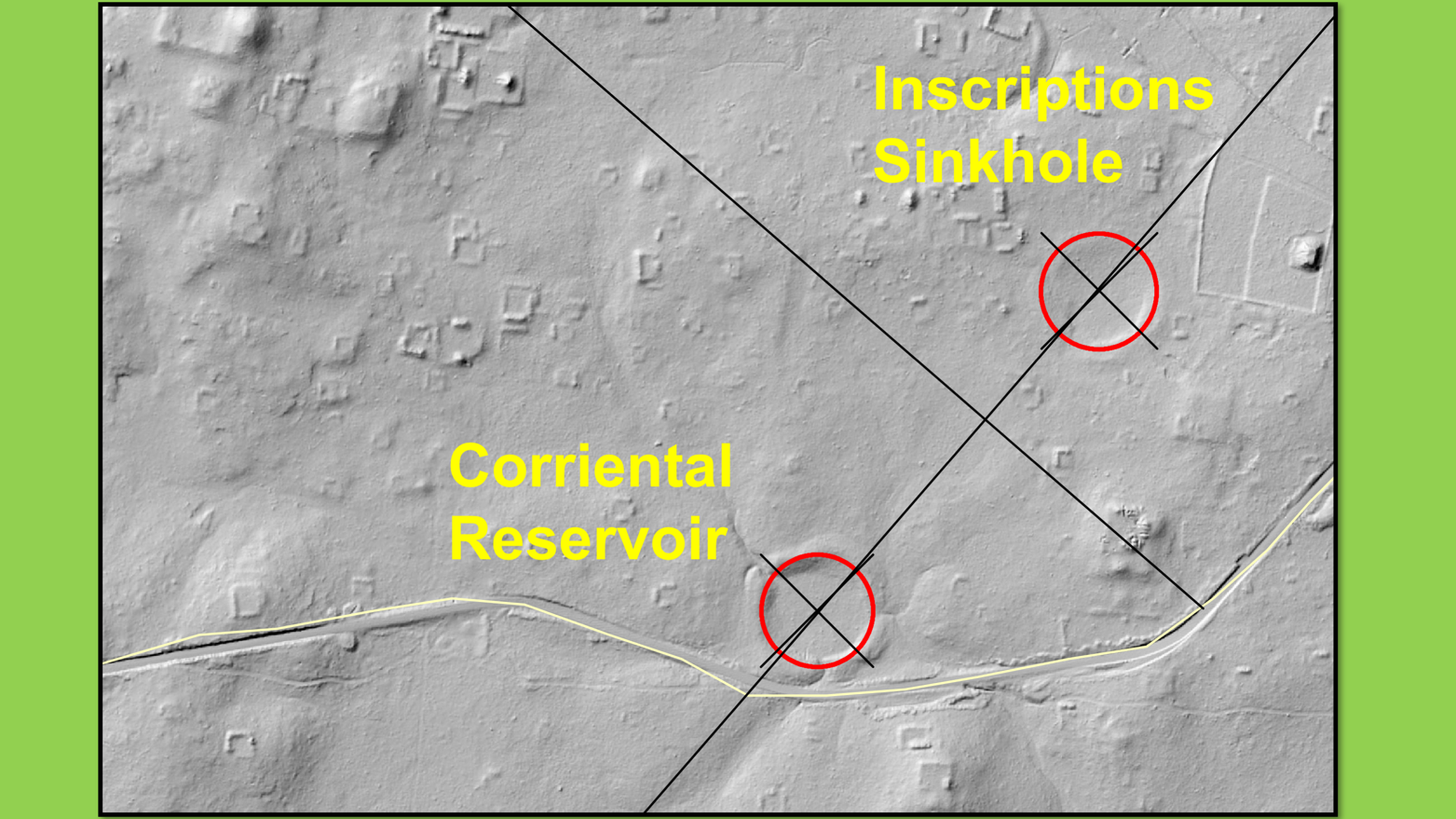


**fig. S15. Comparison of the Corriental Reservoir and the Inscriptions Sinkhole.** A PACUNAM lidar-derived hillshade image. Christopher Carr used ESRI ArcGIS 10.3.1 software ([www.esri.com](http://www.esri.com)) to create a GIS layer georeferenced on the hillshades to make the base map using ESRI ArcGIS 10.3.1 software ([www.esri.com](http://www.esri.com)). Kenneth Barnett Tankersley then used Microsoft PowerPoint for Mac Version 16.41 ([www.microsoft.com](http://www.microsoft.com)) to create this figure.

**References**

S1. Canuto, M. A. et al. Ancient lowland Maya complexity as revealed by airborne laser

scanning of northern Guatemala. Science 361, 6409 (2018).

S2. Callender, D. W. The Reservoirs and Hydraulics of Greater Tikal (University of

Pennsylvania Museum, Philadelphia, 1968).

S3. Harrison, P. D. The marvel of Maya engineering: Water management at Tikal. Expedition

54, 19–26 (2012).

S4. Gallopin, G. G. Water Storage Technology at Tikal, Guatemala (University of Cincinnati,

Cincinnati, 1990).

S5. Scarborough, V. L. Water management as a function of locational and appropriational

movements and the case of the classic Maya of Tikal. In From Political Economy to Anthropology: Situating Economic Life in Past Societies (ed. Stanfield, J. R.) 105–121 (Black Rose Books, Portland, 1994).

S6. Scarborough, V. L. Ecology and ritual: Water management and the Maya. Lat. Am.

Antiq. 9, 135–159 (1998).

S7. Grazioso, S. L. & Scarborough, V. L. Control de Agua por los Antiguos Mayas: El

Sistema Hidrulico de Tikal. Contrib. New World Archaeol. 5, 39–56 (2013).

S8. Scarborough, V. L. & Grazioso, L. G. The evolution of an ancient waterworks system at

Tikal. In Tikal: Paleoecology of an Ancient Maya City (eds Lentz, D. et al.) 16–45 (Cambridge University Press, Cambridge, 2015).

S9. Lane, B., Scarborough, V. L. & Dunning, N. P. At the core of Tikal: Terrestrial sediment

sampling and water management. In Tikal: Paleoecology of an Ancient Maya City (eds Lentz, D. et al.) 46–58 (Cambridge University Press, Cambridge, 2015).

S10. Dunning, N. P. et al. Life on the edge: Tikal in a Bajo landscape. In Tikal: Paleoecology

of an Ancient Maya City (eds Lentz, D. et al.) 95–123 (Cambridge University Press, Cambridge, 2015).

S11. Beach, T. et al. Human and natural impacts on fluvial and karst systems in the Maya

Lowlands. Geomorphology 101, 301–331 (2008).

S12. Chen, P. Y. Table of Key Lines in X-ray Powder Diffraction Patterns of Minerals in

Clays and Associated Rocks (Indiana Geological Survey, Bloomington, 1977).

S13. Dunning, N. P., Beach, T. & Luzzadder-Beach, S. Kax and Kol: Collapse and resilience

in lowland Maya civilization. Proc. Natl. Acad. Sci. 109, 3652–3657 (2012).

S14. Stephens, J. L. Incidents of Travel in Yucatan (Harper & Brothers, Manhatta, 1843).

S15. Dunning, N. P. Lords of the Hills: Ancient Maya Settlement in the Puuc Region, Mexico

(Prehistory Press, Cambridge, 1992).

S16. Maler, T. Península Yucatán (Gebr Mann Verlag, Berlin, 1997).

S17. Lundell, C. L. The Vegetation of the Petén (Carnegie Institution of Washington,

Washington, 1937).

S18. Morley, S. G. The Inscriptions of Petén (Carnegie Institution of Washington, Washington, 1938).

S19. Bullard, W. R. Maya settlement pattern in Northeastern Peten, Guatemala. Am. Antiq.

25, 355–372 (1960).

S20. Dahlin, B. H., Foss, J. & Chambers, M. E. Project Akalches: Reconstructing the natural

and cultural history of a seasonal swamp: Preliminary results. In El Mirador, El Petén, Guatemala: An Interim Report (ed. Matheny, R. T.) 37–58 (Brigham Young University, Provo, 1980).

S21. El Morales-Aguilar, C. A. Sistema Hidráulico de El Mirador, Peten, Guatemala: Una

Perspectiva General (ProyectoArqueol.gico Cuenca Mirador, El Mirador, 2009).

S22. Matheny, R. T. et al. Investigations at Edzná Campeche, Mexico (Brigham Young

University, Provo, 1983).

S23. Huchim Herrera, J. & S.nchez, I. El Sistema Hidra.lico de Uxmal. Boletín Acad. de la

Facult. de Ingeniería 13, 35–44 (1990).

S24. Scarborough, V. L., Connelly, R. & Ross, S. The prehispanic Maya reservoir system at

Kinal, Peten, Guatemala. Ancient Mesoam. 5, 97–106 (1994).

S25. Scarborough, V. L. et al. Water and land at the ancient Maya community of La Milpa.

Lat. Am. Antiq. 6, 98–119 (1995).

S26. Dunning, N. P. et al. Physiography, habitats, and landscapes of the three rivers region. In

Heterarchy, Political Economy, and the Ancient Maya: The Three Rivers Region of the East-Central Yucatan Peninsula (eds Scarborough, V. L. & Dunning, N. P.) 14–24 (University of Arizona, Tucson, 2003).

S27. Weiss-Krejci, E. Ancient Maya rainwater reservoirs in northwestern Belize. Contrib.

New World Archaeol. 5, 85–100 (2013).

S28. Dom.nguezCarrazco, M. & Folan, W. J. Calakmul, M.xico: Aguadas, Bajos, Precipitacin

y Asentamineto en el PetenCampechano. In IX Simposio de Investigaciónes Arqueológicas en Guatemala (eds Laporte, J. P. & Escobedo, H. L.) 171–193 (MuseoNacional de Antropologa y Historia, Mexico, 1996).

S29. Geovannini-Acu.a, H. Rain Harvesting in the Rainforest: The Ancient Maya Agricultural

Landscape of Calakmul, Campeche, Mexico (BAR International Series, Oxford, 2008).

S30. Beach, T. & Dunning, N. An ancient Maya reservoir and dam at Tamarindito, El Peten,

Guatemala. Lat. Am. Antiq. 8, 20–29 (1997).

S31. Wahl, D. et al. A paleoecological record from a late classic Maya reservoir in the north

Peten. Lat. Am. Antiq. 18, 212–222 (2007).

S32. Akpinar-Ferrand, E. et al. Use of Aguadas as water management sources in two southern

Maya lowland sites. Ancient Mesoam. 23, 85–101 (2012).

S33. Dunning, N. P. et al. Harvesting Ha: Ancient water collection and storage in the elevated

interior region of the Maya Lowlands. In Sustainability and Water Management in the Maya World and Beyond (eds Larmon, J. T. et al.) (University Press of Colorado, Boulder, 2020).

S34. Isendahl, C. The weight of water: A new look at pre-hispanic Puuc Maya water

reservoirs. Ancient Mesoam. 22, 185–197 (2011).

S35. Dunning, N. et al. Xcoch: Home of ancient Maya rain gods and water managers. In The

Archaeology of Yucatan: New Directions and Data (ed. Stanton, T. W.) 65–80 (BAR International Series, Oxford, 2014).

S36. Volta, B. et al. Los Rasgos Hidrulicos de Oxpemul, Campeche. Los Investigadores de la

Cultura Maya 21, 265–278 (2013).

S37. Seefeld, N. The Hydraulic System of Uxul: Origins, Functions, and Social Setting

(Archaeopress, Oxford, 2018).

S38. Beach, T. P. et al. A neighborly view: Water and environmental history of the El Zotz

region. In Tikal: Paleoecology of an Ancient Maya City (eds Lentz, D. et al.) 258–278 (Cambridge University Press, Cambridge, 2015).

S39. Thompson, E. H. TheChultuns of Labna (Memoirs of the Peabody Museum, New Haven,

1897).

S40. Zapata Peraza, R. L. Los Chultunes de la regen Serrana de Yucatan. Cuadernos de la

Arquit. Mesoam. 8, 17–27 (1986).

S41. McAnany, P. A. Water storage in the Puuc region of the northern Maya: A key to

population estimates and architectural variability. In Precolumbian Population History in the Maya Lowlands (eds Culbert, T. & Rice, D.) 263–284 (University of New Mexico Press, Mexico, 1990).

S42. Becquelin, P. & Michelet, D. Demografa en la Zona Puuc: el Recursodel M todo. Lat.

Am. Antiq. 5, 289–311 (1994).

S43. Weiss-Krejci, E. & Sabbas, T. The potential role of small depressions as water features in

the central Maya Lowlands. Lat. Am. Antiq. 13, 343–357 (2002).

S44. Brewer, J. L. Householders ad water managers: A comparison of domestic-scale water

management practices at two central Maya Lowlands sites. Ancient Mesoam. 29, 197–217 (2018).

S45. Brewer, J. L. et al. Employing airborne lidar and archaeological testing to determine the

role of small depressions in water management at the ancient Maya site of Yaxnohcah, Campeche, Mexico. J. Archaeol. Sci. Rep. 13, 291–302 (2017).

S46. Bhattacharya, S. S. A. Drinking water contamination and treatment techniques. Appl.

Water Sci. 7, 1043–1067 (2017).

S47. Goldman, N., Pebley, A. R. & Beckett, M. Diffusion of ideas about personal hygiene and

contamination in poor countries: Evidence from Guatemala. Soc. Sci. Med. 53, 53–69 (2001).

S48. Berardi, R. R. et al. Handbook of Nonprescription Drugs (American Pharmacists

Association, Washington, 2006).

S49. Green, R. T. et al. Groundwater contamination in karst terrains. Water Air Soil Pollut.

Focus 6, 157–170 (2006).

S50. Bali, M., Gueddari, M. & Boukchina, R. Removal of contaminants and pathogens from

secondary effluents using intermittent sand filters. Water Sci. Technol. 64, 2038–2043 (2011).

S51. Wang, S. & Peng, Y. Natural zeolites as effective adsorbents in water and wastewater

treatment. Chem. Eng. J. 156, 11–24 (2010).

S52. G.mez-Pompa, A., Salvador Flores, J. & Fern.ndez, M. A. The sacred Cacao groves of

the Maya. Latin Am. Antiq. 1, 247–257 (1990).

S53. Quintana, O. & Wurster, W. W. Ciudades Mayas del Norestedel Petén: Un

Estudio Urbanístico Comparativo (Verlag Philipp von Zabern, Mainz, 2001).

S54. Seefeld, N. The Hydraulic System of Uxul—Origins, Functions, and Social Setting

(Rheinische Friedrich-Wilhelms-Universit, Bonn, 2017).

S55. Guerra, D., Santos, M. & Airoldi, C. Mercury adsorption on natural and organo

functionalized smectites—Thermodynamics of cation removal. J. Braz. Chem. Soc. 20, 4 (2009).

S56. Tankersley, K. B. et al. Volcanic minerals in Chaco Canyon, New Mexico and their

archaeological significance. J. Archaeol. Sci. Rep. 17, 404–421 (2018).

S57. Pendergast, D. Ancient Maya mercury. Science 217, 533–535 (1982).

S58. Blount, D. Geology of the Chiantla Quadrangle, Guatemala (Louisiana State University

and Agricultural & Mechanical College, Baton Rouge, 1967).

S59. Rapp, G. Archaeomineralogy (Springer, New York, 2013).

S60. Blondeau, M. et al. Impact of the cyanobacterium Gloeomargarita lithophora on the

geochemical cycles of Sr and Ba. Chem. Geol. 483, 88–97 (2018).

S61. Carr, R. F. & Hazard, J. E. Map of the Ruins of Tikal, El Peten, Guatemala (University of

Pennsylvania, Philadelphia, 1961).

S62. Carr. C. Tikal Report 11: Map of the Ruins of Tikal, El Petén, Guatemala and

Georeferenced Versions of the Maps Therein. https ://core.tdar.org/proje ct/39092 2 (2013).

S63. Carr, C. et al. Bringing the archaeological maps of Tikal into the era of electronic GIS. In

Tikal: Paleoecology of an Ancient Maya City (eds Scarborough, V. L. & Dunning, N. P.) 59–86 (Cambridge University Press, Cambridge, 2015).
